# FAT10 promotes chemotherapeutic resistance in pancreatic cancer by inducing epithelial-mesenchymal transition via stabilization of FOXM1 expression

**DOI:** 10.1038/s41419-022-04960-0

**Published:** 2022-05-25

**Authors:** Jinfeng Zhu, Jiefeng Zhao, Chen Luo, Zhengming Zhu, Xingyu Peng, Xiaojian Zhu, Kang Lin, Fanqin Bu, Wenjun Zhang, Qing Li, Kai Wang, Zhigang Hu, Xin Yu, Leifeng Chen, Rongfa Yuan

**Affiliations:** 1 Department of General Surgery, the Second Affiliated Hospital of Nanchang University, Nanchang, 330006 Jiangxi Province China; 2 Department of Pathology, the Second Affiliated Hospital of Nanchang University, Nanchang, 330006 Jiangxi Province China; 3 Jiangxi Provincial Clinical Research Center for General Surgery Disease, Nanchang, 330006 Jiangxi Province China; 4 Cancer Center, Renmin Hospital of Wuhan University, Wuhan, 430060 Hubei China

**Keywords:** Ubiquitylation, Oncogenes, Epithelial-mesenchymal transition

## Abstract

Pancreatic cancer (PC) is one of the deadliest malignant tumors, and its resistance to gemcitabine chemotherapy is the primary reason for poor prognosis in patients. Ubiquitin-like protein FAT10 has recently been reported to promote tumor chemotherapy resistance. In this study, the expression of FAT10 in PC was significantly higher than that in adjacent noncancerous tissues. Increased expression of FAT10 in PC was related to a late TNM stage and decreased overall survival. Functional experiments revealed that downregulating the expression of FAT10 inhibits the proliferation and epithelial-mesenchymal transition (EMT) of PC cells, promotes the apoptosis of PC cells, and enhances sensitivity to gemcitabine chemotherapy. In addition, upregulation of FAT10 increased the expression of FOXM1 protein. The effect of downregulating FAT10 was reversed by FOXM1 overexpression, and FOXM1 knockdown inhibited EMT driven by FAT10 overexpression. Mechanistically, FAT10 stabilized the expression of FOXM1 by competing with ubiquitin to bind FOXM1 and inhibiting the ubiquitination-mediated degradation of FOXM1. In conclusion, the FAT10-FOXM1 axis is a pivotal driver of PC proliferation and gemcitabine resistance, and the results provide novel insights into chemotherapy resistance in PC.

## Introduction

Pancreatic cancer (PC) has become one of the most lethal malignant tumors worldwide [1]. Its late clinical diagnosis, strong tumor invasion, and a high degree of metastasis have led to a low resection rate and high recurrence rate. Chemotherapy as gemcitabine (GEM) monotherapy or combination therapy with other chemotherapeutic drugs is the mainstay treatment for PC [2]. However, the resistance of PC cells to GEM has made it difficult to significantly extend the survival time of patients [3]. Therefore, it is of great significance to explore methods for improving the chemotherapy sensitivity of PC and identifying new targets of chemotherapy sensitivity for the treatment of PC.

Human leukocyte antigen F-associated transcript10 (FAT10), a member of the ubiquitin-like (UBL) protein family, is involved in cellular immune/inflammatory mediation, apoptosis, cell cycle regulation, signal transduction, etc. [4–6]. FAT10 is a special ubiquitin-like protein that directly mediates the ubiquitin-independent proteasome degradation of substrates. Interestingly, our previous studies were the first to confirm that FAT10 has the function of stabilizing substrate in different cancer cells [7, 8]. Furthermore, our study has also showed that FAT10 could exert the degradation and stabilization functions in tumor cells simultaneously [9]. The role of FAT10 in the occurrence and development of tumors has been highlighted recently. For example, the expression of *FAT10* is upregulated in various cancers, such as hepatocellular carcinoma (HCC), gastrointestinal cancer, gynecological cancer, osteosarcoma, and bladder cancer [10–16]. As an independent prognostic factor, FAT10 promotes the progression of hepatitis B virus-related HCC [17]. It promotes the invasion and metastasis of HCC cells by upregulating the expression of human homeobox B9 through the β-catenin/TCF4 pathway [8]. In addition, we previously found that FAT10 overexpression promotes the progression of HCC by affecting the inconsistent expression of WISP1 protein and mRNA [9].

FAT10 also contributes to chemotherapeutic resistance. When FAT10 expression is downregulated, HCC cell apoptosis and the sensitivity of HCC to 5-fluorouracil are increased [17]. Additionally, decreasing the expression of FAT10 reduces chemotherapy resistance in non-small cell lung cancer [13]. Moreover, upregulating the expression of FAT10 promotes cisplatin resistance in bladder cancer [18]. FAT10 expression is upregulated in PC [19], but its role and mechanism in chemotherapeutic resistance in PC remain unclear.

Epithelial-mesenchymal transition (EMT) is the biological process wherein the epithelial cell phenotype transforms into the mesenchymal cell phenotype [20, 21]. Studies have shown that EMT plays an important role in the chemotherapy resistance of PC and other malignant tumors and is considered to be an important mechanism of tumor chemotherapy resistance [22–26]. Forkhead box M1 (FOXM1) plays a vital role in EMT [27, 28]. FOXM1 belongs to the forkhead transcription factor family, is located on chromosome 12p13.3, and comprises 10 exons that span approximately 25 kb [29]. It regulates cell proliferation, cell differentiation, DNA repair, cell aging, apoptosis, and tissue homeostasis [30]. It regulates EMT and leads to the progression and chemotherapeutic resistance of ovarian cancer, nasopharyngeal cancer, cervical cancer, and other cancers [31–36]. Moreover, studies have found that FOXM1 promotes tumor metastasis by inducing EMT in tumor cells in HCC and PC [37–40]. However, whether FOXM1-mediated EMT is the cause of chemotherapeutic resistance in PC and its upstream regulatory mechanism have not been fully elucidated.

In this study, we investigated the role and mechanism of FAT10 in the chemotherapeutic resistance of PC to GEM. First, we demonstrated that FAT10 is highly expressed in both PC tissues and drug-resistant cell lines. Second, we confirmed that FAT10 makes PC cells resistant to GEM by upregulating FOXM1 to induce EMT. Finally, we revealed the molecular mechanism by which FAT10 and ubiquitin competitively bind to FOXM1 and stabilize FOXM1 protein expression.

## Results

### FAT10 is overexpressed in PC and is related to poor clinical prognosis in patients

**Fig. 1:**
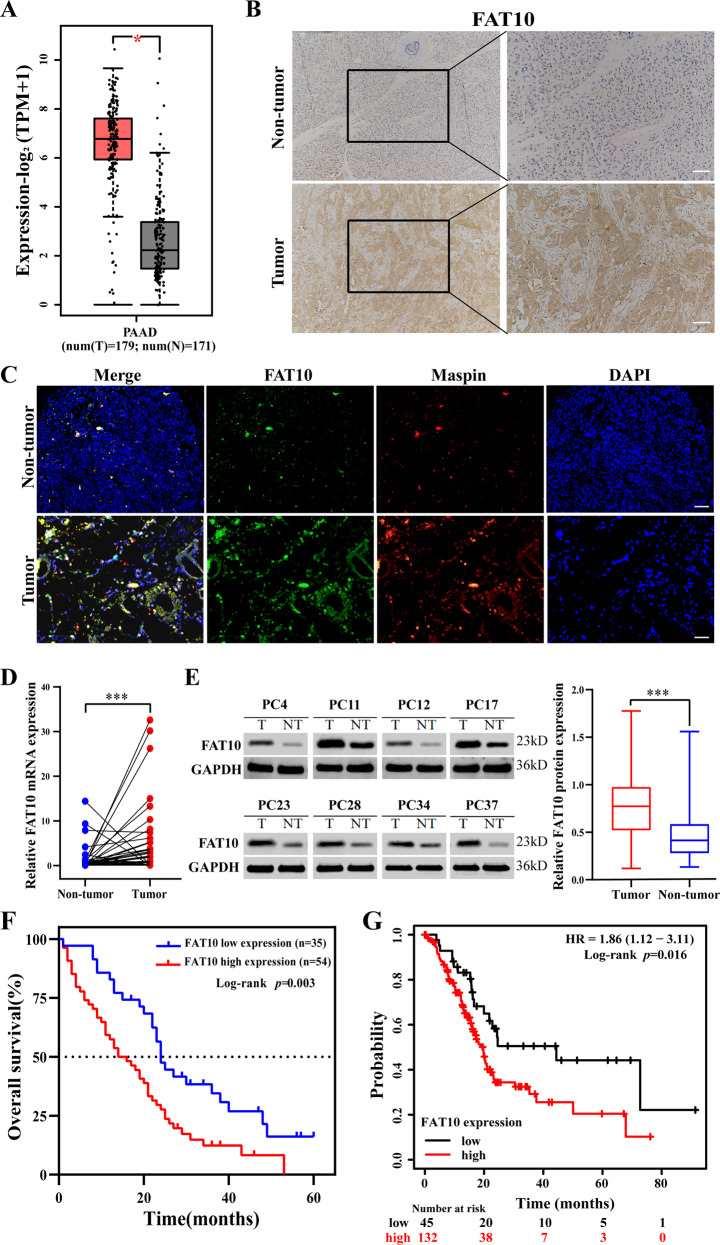
High FAT10 expression is associated with poor prognosis in patients with PC. **A** The GEPIA2 server was used to analyze the expression of *FAT10* in PAAD (T, tumor; N, nontumorous tissues; *p < 0.05). **B** Representative IHC image showing the increase in FAT10 protein levels in PC tissue (magnification: 100×, inset magnification: 200×). Scale bar, 200 μm. **C** Immunofluorescence localization of FAT10 protein expression in PC and paracancerous tissues using the anti-FAT10 antibody (1:100, green) and anti-Maspin antibody (1:100, red), followed by DAPI nuclear counterstaining (blue). Merged images of FAT10 (green) and Maspin (red) with DAPI (blue) are also shown. Scale bar, 100 μm. **D** qRT–PCR analysis of FAT10 mRNA levels in PC tissues and corresponding adjacent tissues (*n* = 40, *p* = 0.001; Wilcoxon signed-rank test). GAPDH was used as a loading control. **E** Western blot analysis of FAT10 protein expression in PC tissues and corresponding adjacent noncancerous tissues (*n* = 40; Student’s t-test). GAPDH was used as a loading control. **F** Kaplan–Meier survival curve of the relationship between FAT10 high and low expression groups and the prognosis of PC patients (*n* = 89, *p* = 0.003; Log-rank test). **G** Kaplan–Meier survival curves of prognosis-related *FAT10* expression in the Kaplan–Meier plotter cohort (*n* = 177, *p* = 0.016; Log-rank test).

To explore the expression and significance of FAT10 in PC, we first evaluated the expression of FAT10 in PC and normal tissues using the GEPIA2 server. The Cancer Genome Atlas (TCGA) and the Broad Genotype-Tissue Expression (GTEx) portal results revealed significant overexpression of FAT10 (*p* < 0.05) in pancreatic adenocarcinoma compared to noncancerous tissue (Fig. 1A). Next, we analyzed 89 samples of PC and adjacent tissues. Immunohistochemical (IHC) analysis showed that FAT10 was overexpressed in 60.67% (54/89) of PC samples (Fig. 1B). Co-localization immunofluorescence analysis of the tumor cell marker Maspin [41] and FAT10 showed that FAT10 was overexpressed in PC tissues compared with the stromal component (Fig. 1C). In addition, we analyzed 40 pairs of freshly collected specimens. qRT-PCR and western blot results demonstrated that FAT10 mRNA and protein were overexpressed in PC compared with the corresponding neighboring noncancerous tissues (*p* < 0.001, Fig. 1D, E). Subsequently, we examined the correlation between FAT10 expression and clinicopathological characteristics of patients with PC. High FAT10 expression was closely related to a late TNM stage (*p* = 0.030) but was not significantly related to age, sex, tumor size, degree of differentiation, nerve invasion, or lymph node metastasis (Table 1). According to Kaplan–Meier survival analysis, the overall survival (OS) of patients with PC with low FAT10 expression was better than that of patients with PC with high FAT10 expression (*p* = 0.003, Fig. 1F). Moreover, an analysis of the effect of FAT10 on OS in 177 pancreatic cancer cases in public databases by using Kaplan–Meier Plotter showed that patients with high FAT10 expression had a worse prognosis (*p* = 0.016, Fig. 1G), consistent with our conclusion. In summary, these results indicate that FAT10 is upregulated in PC and is related to prognosis in PC patients.

**Table 1:** The relationship between FAT10 expression and clinicopathological characteristics in PC patients.

			FAT10 expression		
Features		*n*	Low	High	*p*-value
All cases		89	35	54	
Age (years)					0.851
	<60	29	11	18	
	≥60	60	24	36	
Gender					0.152
	Man	45	21	24	
	Female	44	14	30	
Tumor size (cm)					0.643
	≤3	51	19	32	
	>3	38	16	22	
Lymph node metastasis					0.051
	Negative	42	21	21	
	Positive	47	14	33	
TNM stage					***0.030***
	I and II	68	31	37	
	III and IV	21	4	17	
Distant metastasis					0.195
	Negative	73	31	42	
	Positive	16	4	12	
Perineural invasion					0.126
	Negative	25	13	12	
	Positive	64	22	42	

Bold Italic values are statistically significant, *p* < 0.05.

### FAT10 overexpression is associated with GEM resistance in PC cells

**Fig. 2:**
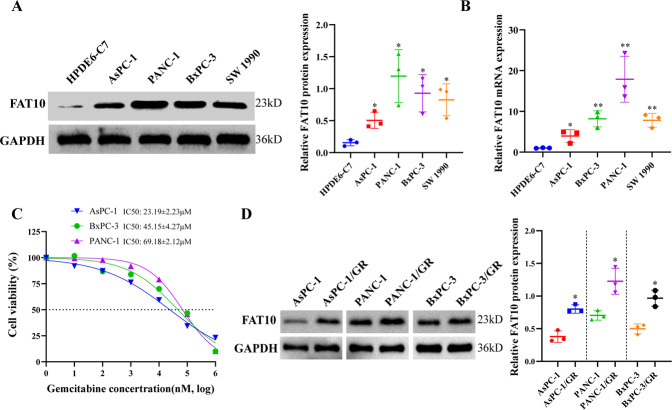
FAT10 expression is correlated with chemoresistance of PC cells to GEM. **A** Protein expression of FAT10 in noncancerous pancreatic ductal epithelial cells and PC cell lines was analyzed by western blotting. **B** The mRNA expression of FAT10 in normal pancreatic ductal epithelial cells and PC cell lines was analyzed by qRT–PCR. **C** Viabilities of PC cells were determined in response to different concentrations of GEM. Inhibition curves were fitted by nonlinear regression, and GEM IC50s were calculated using GraphPad Prism 8 software. **D** Western blotting was used to analyze expression levels of FAT10 in GEM-resistant (GR) PC cells. Data represent the mean ± SD of triplicate experiments and were statistically analyzed with Student’s t-test, *p < 0.05, **p < 0.01.

To explore the relationship between FAT10 expression and the sensitivity of PC cells to GEM in vitro, we first determined the expression levels of FAT10 in noncancerous human pancreatic ductal epithelial cells and PC cells using qRT-PCR and western blot analyses. The results showed that FAT10 is highly expressed in PC cells, with PANC-1 cells expressing the highest levels and AsPC-1 cells expressing the lowest levels (Fig. 2A, B). Subsequently, we analyzed the viability of PC cells exposed to different concentrations of GEM and obtained the corresponding IC50 (Fig. 2C). Interestingly, cell lines with higher FAT10 expression were more resistant to GEM, and FAT10 expression was higher in GEM-resistant (GR) PC cell lines than in the PC parent cell line (Fig. 2D). The above data indicate that FAT10 is highly expressed in PC cell lines, and the resistance of PC cells to GEM chemotherapy is related to the increased expression of FAT10.

### Inhibition of FAT10 increases the chemotherapeutic sensitivity of PC to GEM in vivo and in vitro

**Fig. 3:**
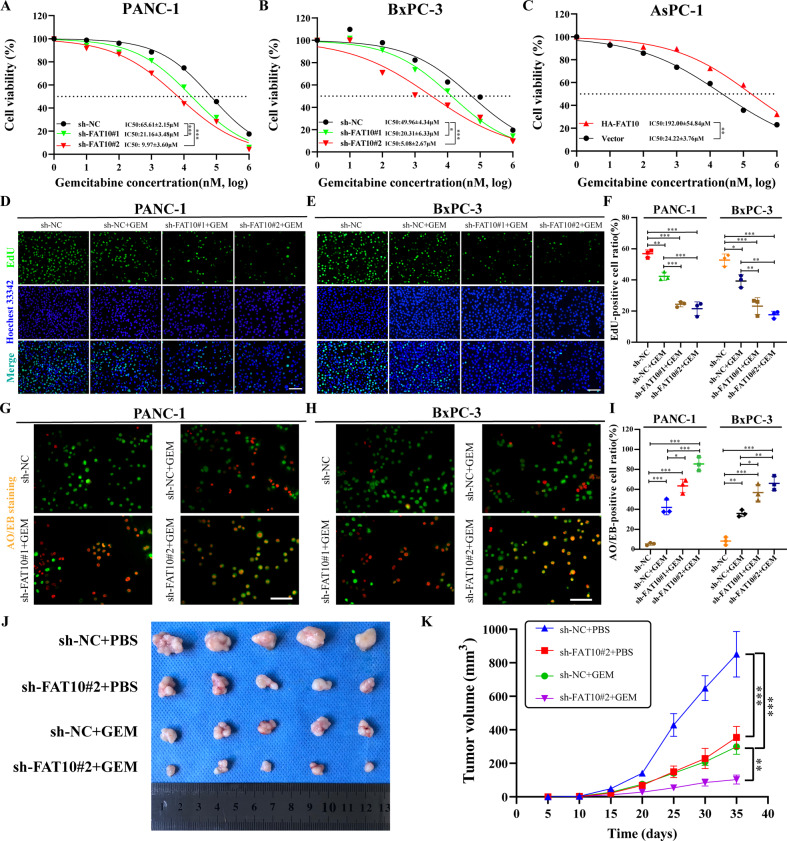
Inhibition of FAT10 increases the chemotherapeutic sensitivity of PC to GEM. **A**–**C** The IC50 of PC cells (FAT10 knockdown or overexpression) exposed to different concentrations of GEM was determined by cell viability experiments. **D**–**F** The effect of knocking down FAT10 on the proliferation rate of PC cells treated with GEM was detected by EdU staining. **G**–**I** The apoptosis rate of PC cells with FAT10 knockdown was detected by AO/EB staining after GEM treatment. Data represent the mean ± SD of triplicate experiments and were statistically analyzed by one-way ANOVA. **J** Representative images of different groups of tumors removed from mice are shown. **K** Growth curve showing changes in tumor volume in mice from different groups. Growth was assessed every 5 days beginning from the day of injection and during GEM treatment. At the end of the experiment, the tumor was dissected and photographed, and the tumor volume (V) was calculated as follows: *V* = 0.52 × length × width^2^ and analyzed by one-way ANOVA. *p < 0.05, **p < 0.01, ***p < 0.001.

To address the potential role of FAT10 in the sensitivity of PC to GEM, we conducted the following experiment. In vitro, we transfected FAT10 shRNA and plasmid into PC cells and verified the transfection effect using qRT-PCR and western blot analyses (Fig. S1A–D). Subsequently, we exposed PC cells to different concentrations of GEM. Using cell viability experiments, we found that inhibiting the expression of FAT10 increased the sensitivity of PC cells to GEM (all *p* < 0.05, Fig. 3A, B). Conversely, increasing the expression of FAT10 reduced the sensitivity of PC cells to GEM (*p* < 0.01, Fig. 3C). In addition, we verified this result using EdU cell proliferation and AO/EB cell apoptosis experiments. Downregulating the expression of FAT10 enhanced the inhibitory effect of GEM on PC cell proliferation (Fig. 3D–F) and the promotion of PC cell apoptosis (Fig. 3I), while upregulating FAT10 attenuated these effects (Fig. S2A–D).

In vivo, we injected mice with PC cells with stable knockdown of FAT10 and regularly treated them with intraperitoneal GEM. After 35 days, compared to the sh-NC group, the tumor volume of the nude mice in the sh-FAT10#2 and the sh-NC + GEM groups was reduced, while the tumor volume of the nude mice in the sh-FAT10#2 + GEM group was significantly reduced (all *p* < 0.01, Fig. 3J, K). These data collectively indicate that inhibition of FAT10 improves the chemotherapeutic sensitivity of PC cells to GEM.

### FAT10 regulates EMT to promote chemotherapeutic resistance in PC cells

**Fig. 4:**
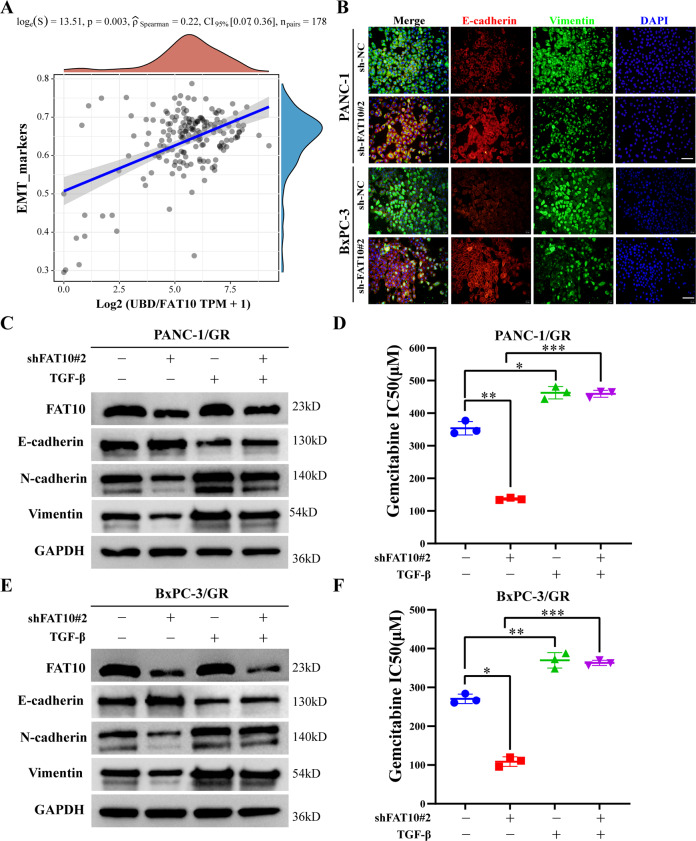
FAT10 regulates EMT to promote chemotherapeutic resistance in PC cells. **A** Spearman correlation analysis of the correlation between *FAT10* (*UBD*) and EMT pathway score. *FAT10* expression is represented by the abscissa, and the EMT pathway score is represented by the ordinate. A density curve to the right represents the trend in the distribution of pathway scores, a density curve to the upper part represents the trend in the distribution of gene expression. The top part shows the *p*-value, correlation coefficient, and correlation calculation method. **B** Immunofluorescence analysis of the effect of inhibiting FAT10 on the expression of EMT-related proteins (E-cadherin and Vimentin) in PC cells. Scale bar, 50 μm. **C**–**F** GEM-resistant PC cell lines (PANC-1/GR and BxPC-3/GR) were transfected with interfering plasmid sh-FAT10#2 and or EMT activator (TGF-β, 10 ng/mL) for 48 h. **C**, **E** Western blot analysis was used to observe the expression of EMT-related proteins in each treatment group. **D**, **F** Cell viability experiments were used to calculate the half-inhibitory concentration (IC50) of GEM-treated PC-resistant cells in each treatment group. Data represent the mean ± SD of triplicate experiments and were statistically analyzed by one-way ANOVA. *p < 0.05, **p < 0.01, ***p < 0.001.

EMT is one of the reasons for drug resistance in many solid tumors [42]. We speculate that FAT10 may be involved in regulating EMT in PC cells, thereby promoting chemotherapy resistance in PC cells. To test this hypothesis, we first obtained RNA-seq data and corresponding clinical information from 178 cases of PC samples from TCGA database, and our ssGSEA analysis found that FAT10 expression was positively correlated with the EMT pathway (*p* = 0.003, Fig. 4A). Furthermore, to clarify the relationship between FAT10 and EMT signaling pathway, we conducted an immunofluorescence analysis and observed the change in EMT marker expression by reducing FAT10 expression. Our results show that decreased FAT10 expression leads to increased expression of E-cadherin protein and decreased expression of Vimentin protein, suggesting that decreased FAT10 expression can inhibit EMT occurrence in PC cells (Fig. 4B). To further confirm that FAT10 affects the chemotherapeutic resistance of PC cells through EMT, we reduced the expression of FAT10 in drug-resistant pancreatic cancer cells and added an EMT activator to observe changes in the sensitivity of drug-resistant PC cells to chemotherapy. We found that decreasing FAT10 expression could increase the sensitivity of drug-resistant PC cells, but the addition of the EMT activator blocked this process (Fig. 4C–F). These results demonstrated that FAT10 regulates EMT to affect the chemotherapeutic sensitivity of PC.

### FAT10 regulates EMT through FOXM1

**Fig. 5:**
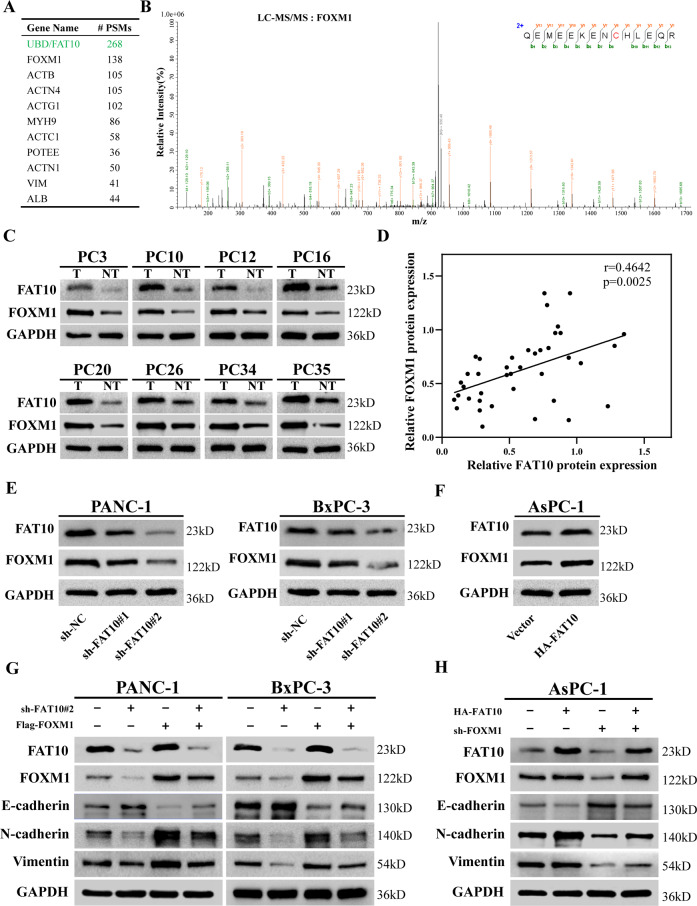
FAT10 regulates EMT through FOXM1. **A** Top 10 proteins co-precipitated with FAT10 analyzed by LC-MS/MS. #PSMs, peptide spectrum matches. **B** Mass spectrum showing unique peptides of FOXM1 identified by 2D-LC-MS/MS from the protein lysates prepared from PANC-1 cells following immunoprecipitation with anti-FAT10. **C** Representative western blot analysis of FAT10 and FOXM1 protein expression in PC and paired paracancerous tissues (T, tumor; NT, non-tumor tissue). **D** Scatter plots showing a positive correlation between FAT10 and FOXM1 protein expression levels in 40 PC samples (*n* = 40, *r* = 0.4642, *p* = 0.0025, Pearson test). **E**, **F** Western blot analyses were used to detect FAT10 and FOXM1 protein expression in cells stably transfected with the shFAT10 or HA-FAT10 plasmid. **G** Western blot analysis confirming FAT10 silencing and FOXM1 restoration and their effects on EMT-related proteins. **H** Western blot analysis showing the levels of FAT10 overexpression and FOXM1 inhibition and their effects on EMT-related proteins.

To further explore how FAT10 regulates EMT and influences the chemotherapeutic sensitivity of PC cells, we identified proteins binding with FAT10 in PC cells by co-immunoprecipitation (IP) mass spectrometry and found that FOXM1 and FAT10 bind each other (Fig. 5A, Table S1). The secondary mass spectrum of FOXM1 is shown in Fig. 5B. FOXM1 is an important molecule that promotes EMT [43, 44]. Therefore, we investigated whether FAT10 regulates the EMT process of PC cells through FOXM1. First, we assessed the correlation between FAT10 expression and FOXM1 expression in PC tissues. Western blot analysis showed that FAT10 and FOXM1 proteins are both abundantly expressed in PC tissues, and their expression levels were positively correlated (*r* = 0.4642, *p* = 0.0025, Fig. 5C, D). We also found that the protein expression of FOXM1 decreased when FAT10 expression was downregulated and vice versa (Fig. 5E, F).

To clarify whether FAT10 regulates EMT through FOXM1, we first upregulated the expression of FOXM1 in PC cell lines with stable knockdown of FAT10 and then analyzed changes in the EMT of PC cells using western blot analysis. Upregulation of FOXM1 expression reversed the inhibitory effect of FAT10 downregulation on EMT (Fig. 5G). In contrast, downregulation of FOXM1 expression in PC cells with stable overexpression of FAT10 inhibited the FAT10-induced EMT process (Fig. 5H). The above experimental results indicate that FAT10 regulates EMT through FOXM1.

### FAT10 stabilizes the expression of FOXM1 by affecting the ubiquitination levels of FOXM1 in PC cells

**Fig. 6:**
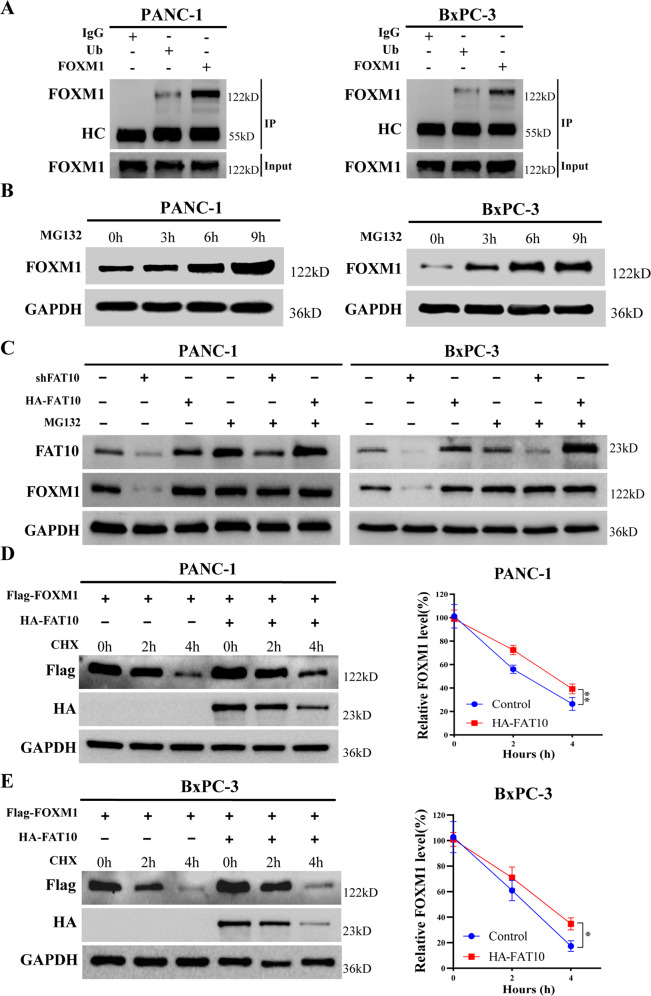
FAT10 increases FOXM1 protein levels by inhibiting the ubiquitination and degradation of FOXM1 in PC cells. **A** Co-IP was used to detect the interaction between FOXM1 and ubiquitin in PANC-1 and BxPC-3 cells. HC, heavy chain. **B** With MG132 (10 μM) added to PANC-1 and BxPC-3 cells, western blotting was used to detect protein levels of FOXM1 at different times. **C** MG132 (10 μM) was added to PANC-1 and BxPC-3 cells while the expression of FAT10 was altered. Western blotting was used to detect protein expression levels of FOXM1. **D** PANC-1 and BxPC-3 cells were treated with CHX (20 μM) for a specified time with or without the addition of the FAT10 overexpression plasmid, and FOXM1 protein levels were detected by western blotting. Data represent the mean ± SD of triplicate experiments and were statistically analyzed with Student’s t-test, *p < 0.05, **p < 0.01.

Although FAT10 increased the protein expression levels of FOXM1, it did not affect the mRNA levels of FOXM1 (Fig. S3). Studies have reported that the FOXM1 protein is degraded through the ubiquitin-proteasome(UPS) pathway [45]. It is unclear whether FAT10, as a ubiquitin-like protein, regulates FOXM1 ubiquitination and degradation. To test if FAT10 regulates FOXM1 expression by inhibiting FOXM1 ubiquitination-mediated degradation, we conducted a co-IP assay that showed the direct binding between endogenous FOXM1 and ubiquitin in PANC-1 and BxPC-3 cells (Fig. 6A). Moreover, the addition of the proteasome inhibitor MG132 to PC cells resulted in the accumulation of endogenous FOXM1 over time (Fig. 6B). This confirms that the ubiquitin-proteasome pathway mediates the degradation of FOXM1 protein in PC cells.

Next, we added the proteasome inhibitor MG132 to PANC-1 and BxPC-3 cells transfected with shFAT10 and HA-FAT10 plasmids. After adding MG132, upregulation or downregulation of FAT10 expression had no significant effect on FOXM1 protein expression in PC cells (Fig. 6C). Subsequently, we added cycloheximide (CHX) to PANC-1 and BxPC-3 cells transfected with Flag-FOXM1 plasmid and HA-FAT10 plasmid to block protein synthesis. Western blot analysis showed that the half-life of exogenous FOXM1 in PC cells overexpressing FAT10 was significantly increased (Fig. 6D, E). These results confirmed that FAT10 participates in the ubiquitin-mediated degradation of FOXM1 protein to stabilize its expression.

### FAT10 inhibits FOXM1 protein ubiquitination degradation by competing with ubiquitin to bind to FOXM1

**Fig. 7:**
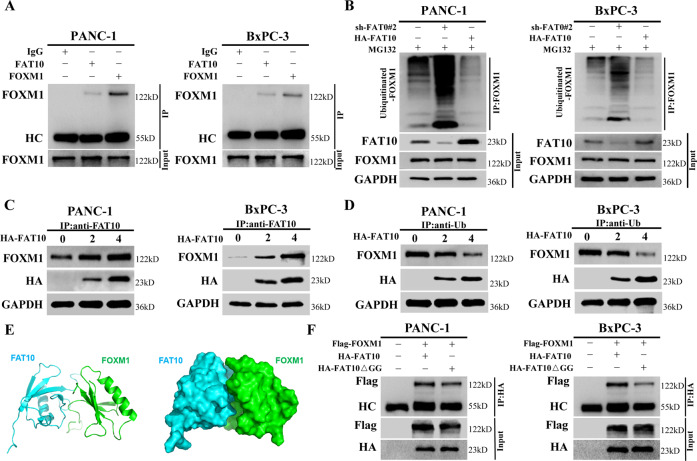
FAT10 competes with ubiquitin to bind and stabilize FOXM1. **A** Co-IP was used to detect the interaction between FOXM1 and FAT10 in PANC-1 and BxPC-3 cells. HC, heavy chain. **B** MG132 (10 μM) was added to PANC-1 and BxPC-3 cells, shFAT10 or HA-FAT10 plasmid was transfected at the same time, and then co-IP was used to detect the levels of ubiquitin bound to FOXM1 protein. **C** PANC-1 and BxPC-3 cells were transfected with different amounts of HA-FAT10 plasmid, and the level of FAT10 binding to FOXM1 protein was detected by co-IP. **D** PANC-1 and BxPC-3 cells were transfected with different amounts of HA-FAT10 plasmid, and the level of Ub binding to FOXM1 protein was detected by co-IP. **E** Docking conformation of the first ranking score. Three-dimensional structure of FAT10 and FOXM1. FAT10 is shown in green. FOXM1 is shown in cyan. **F** The Flag-FOXM1 plasmid was transfected into PANC-1 and BxPC-3 cells, the HA-FAT10 or HA-FAT10ΔGG plasmid was transfected at the same time, and then the protein level of Flag-FOXM1 bound to the HA-tagged protein was detected by co-IP. HC, heavy chain.

To further clarify the role of FAT10 in the degradation of FOXM1, we first needed to determine whether FAT10 and FOXM1 proteins directly interact. Co-IP assay results suggested a possible interaction between FAT10 and FOXM1 proteins in PC cells (Fig. 7A). We previously found that FAT10 stabilizes the substrate by competing with ubiquitin (Ub) and ultimately reducing substrate ubiquitination [7, 8]. Therefore, we tested if FAT10 competes with Ub to bind FOXM1 and inhibits the ubiquitination and degradation of FOXM1 in PC cells by treating PANC-1 and BxPC-3 cells with MG132 to inhibit proteasome-mediated protein degradation. Co-IP assay results showed that FAT10 knockout increased the ubiquitination levels of endogenous FOXM1, while FAT10 overexpression decreased the ubiquitination levels of endogenous FOXM1 (Fig. 7B). These results indicate that FAT10 stabilizes the expression of FOXM1 by inhibiting the ubiquitination of FOXM1 in PC.

Next, we transfected PANC-1 and BxPC-3 cells with HA-FAT10 plasmids of different concentrations. Co-IP assay results showed that as exogenous levels of FAT10 increased, levels of the FAT10-FOXM1 complex increased, while levels of the Ub-FOXM1 complex gradually decreased (Fig. 7C, D). These results confirm that FAT10 and Ub competitively bind to FOXM1 and that overexpression of FAT10 reduces the formation of the Ub-FOXM1 complex, thereby increasing FOXM1 expression.

Docking analysis revealed the binding interactions between FAT10 and FOXM1 (Fig. 7E). The two glycine residues (GG) at the C-terminal of FAT10 play a role in substrate degradation [46, 47], while FAT10 mutated with two glycine residues at the C-terminal can still bind the substrate and stabilize the expression of the substrate protein [9]. Co-IP assay of the binding of HA-FAT10Δ GG and FOXM1 showed that HA-FAT10△GG could still bind Flag-FOXM1 (Fig. 7F). Overall, FAT10 promotes chemotherapeutic resistance in PC by inducing EMT and stabilizing FOXM1 expression.

## Discussion

PC is currently one of the deadliest malignant tumors [48]. Unfortunately, its high chances of becoming resistant to chemotherapeutic drugs [49] have reduced the efficacy of GEM in treating PC. Therefore, it is vital to better understand the mechanism of chemoresistance in PC and find new targets for improving the chemosensitivity of PC cells.

FAT10 is a ubiquitin-like protein (UBL) involved in various basic biological processes [4–6]. Recent studies have also confirmed that FTA10 is related to tumor development and drug resistance. For example, Deng et al. demonstrated that FAT10 induces glycolysis to promote the proliferation of osteosarcoma cells [15]. Liu et al. reported that FAT10 promotes the progression of hepatitis B virus-related HCC through the Akt/GSK3β pathway. At the same time, downregulating FAT10 increases the chemotherapeutic sensitivity of 5-FU to HCC [17]. Xue et al. reported that in cisplatin- and carboplatin-resistant non-small cell lung cancer cells, the protein levels of FAT10 are increased, and knocking down FAT10 reduces the drug resistance of these cells [13]. Li et al. found that FAT10 promotes the formation of cancer-initiating cells and cisplatin resistance in bladder cancer [18]. Sun et al. found that FAT10 is highly expressed in PC tissues and is an independent prognostic factor in patients with PC [19]. However, no information is currently available on the role or molecular mechanism of FAT10 in the chemotherapeutic resistance of PC. We believe that the present study is, to the best of our knowledge, the first study to explore the role of FAT10 in this area. In this study, we confirmed the overexpression of FAT10 in PC tissues and cells and that the high FAT10 expression is associated with poor prognosis in PC. Furthermore, we found that FAT10 is highly expressed in GEM-resistant PC cell lines and that the expression of FAT10 is positively correlated with GEM resistance in PC cells. In vivo and in vitro experiments revealed that reducing the expression of FAT10 inhibits the proliferation of PC cells, makes them more sensitive to GEM treatment, and promotes PC cell apoptosis, thereby indicating that FAT10 may serve as a biomarker of the chemotherapeutic sensitivity of PC.

EMT plays a vital role in cancer chemotherapeutic resistance [50, 51]. For example, acetylation of KLF5 can maintain EMT and carcinogenicity, thereby promoting chemoresistance in prostate cancer [52]. Li et al. found that S100A16-induced EMT promotes metastasis and gemcitabine resistance in human PDAC cells [53]. In addition, studies have shown that FAT10 can induce EMT in multiple tumors. For example, it has been reported that downregulation of FAT10 significantly inhibits the metastatic ability and EMT of breast cancer cells [14]. Li et al. demonstrated that upregulation of FAT10 protein expression in UMUC-3 bladder cancer cells promotes EMT [18]. Liu et al. reported that FAT10 induces EMT and promotes the invasion of HCC [17]. However, whether FAT10 also regulates EMT-mediated chemotherapy resistance in PC is unclear. Our experimental data revealed that when the expression of FAT10 is inhibited, the process of EMT is reversed in GEM-resistant PC cell lines, but in the presence of an EMT activator, this reversal was not seen. Therefore, these results demonstrated that FAT10 regulates EMT to affect the chemotherapeutic sensitivity of PC.

FOXM1 is known to critically regulate EMT [54, 55]. Cao et al. reported that osteopontin activates the integrin αvβ3-Akt/Erk-FOXM1 cascade in a paracrine manner to promote EMT and cancer stem cell-like properties of PC cells [27]. Similarly, Huang et al. found that FOXM1 induces EMT and metastasis in PC through the direct transcriptional activation of caveolin-1[37]. However, the upstream mechanism by which FOXM1 regulates the chemotherapeutic resistance of PC has not been fully elucidated. In this study, we first observed that both FAT10 and FOXM1 have high protein expression levels in PC, and the expression levels were positively correlated; knocking down FAT10 reduced the expression of FOXM1 protein. Using in vitro experiments, we observed that the upregulation of FOXM1 rescues changes in EMT-related protein levels caused by FAT10 knockdown and that downregulation of FOXM1 reverses the FAT10 overexpression-induced EMT. These results indicate that FOXM1 is a key factor by which FAT10 induces EMT in PC cells.

Next, we examined the mechanism by which FAT10 regulates FOXM1. FAT10 is the only UBL modifier that directly targets the substrate protein to be degraded by the 26S proteasome [56]. FAT10 targets degradable proteins, such as p62/SQSTM1, USE1, UBE1, APOL1, JunB, and OTUB1 [46, 47, 57–60]. However, we previously found for the first time that FAT10 also stabilizes substrate proteins [7–9]. Therefore, FAT10 functions to both degrade and stabilize the substrate protein. At the same time, ubiquitination is also an important method of regulating the expression of FOXM1 protein [61, 62]. Thus, we speculate that FAT10 may stabilize the expression of FOXM1 by affecting the ubiquitination levels of FOXM1. First, our results confirmed that FAT10 directly interacts with FOXM1 protein in PC cells and that degradation of FOXM1 protein occurs through the ubiquitin-proteasome pathway. When MG132 was used to inhibit proteasome function, the downregulation or upregulation of FAT10 protein expression did not affect FOXM1 protein expression. In contrast, when CHX was used to block protein synthesis, the degradation rate of FOXM1 in PC cells upregulated by exogenous FAT10 slowed down, indicating that FAT10 is involved in the degradation of FOXM1 by the proteasome. Finally, we found that as exogenous FAT10 increases, levels of the FAT10-FOXM1 complex also increase, while levels of the Ub-FOXM1 complex gradually decrease, demonstrating that FAT10 and Ub competitively bind to the FOXM1 protein, thereby stabilizing the FOXM1 protein. Therefore, we believe that the mechanism by which FAT10 stabilizes the FOXM1 protein may be related to FAT10 and ubiquitin antagonizing FOXM1.

**Fig. 8:**
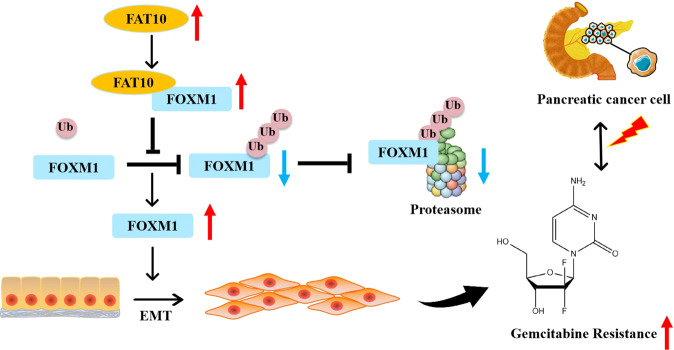
Diagram of the regulatory mechanism of FAT10 promoting chemoresistance in PC. Proposed model by which FAT10 stabilizes the FOXM1 protein by competing with ubiquitin to bind FOXM1, thereby promoting EMT and chemotherapeutic resistance in PC cells.

In conclusion, we demonstrated that FAT10 promotes EMT-mediated chemotherapeutic resistance in PC by stabilizing the expression of FOXM1. We also demonstrated that FAT10 stabilizes FOXM1 by competing with Ub to bind FOXM1 and inhibiting its ubiquitination degradation, thereby stabilizing the FOXM1 protein (Fig. 8). Furthermore, FAT10 regulates the EMT process of PC and ultimately increases the chemotherapeutic resistance of PC to GEM. These results suggest that FAT10 contributes to the chemotherapeutic resistance of PC by enhancing FOXM1-mediated EMT. Thus, targeting the FAT10/FOXM1 axis could be a potential strategy for PC therapy.

## Materials and methods

### Bioinformatics analysis

The differential expression of FAT10 in PC and normal tissues was assessed using Gene Expression Profiling Interactive Analysis 2 (GEPIA2, http://gepia2.cancer-pku.cn/). Kaplan–Meier analysis of FAT10 (UBD) in pancreatic cancer was conducted using Kaplan–Meier Plotter (http://kmplot.com/analysis/). RNA-seq data (level 3) and corresponding clinical information of 178 pancreatic cancer samples were obtained from TCGA database (https://portal.gdc.cancer.gov/). The gene set [63] contained in the relevant pathway was analyzed using the GSVA package, with the parameter method = ‘ssgsea.’ The enrichment score of each sample on each pathway was calculated according to the ssGSEA algorithm [64] to obtain the sample, and the connection between the pathways. Finally, the correlation between *FAT10* expression and EMT pathway score was analyzed by Spearman correlation. Analytical methods and R software packages were used with R software version v4.0.3.

### Tissue specimens

PC and corresponding adjacent tissues were derived from patients with PC who underwent radical surgical resection at the Second Affiliated Hospital of Nanchang University from January 2012 to January 2016. All specimens were pathologically diagnosed as PC. IHC analysis was performed on 4% polymethanol-fixed and paraffin-embedded PC tissue samples. Additional freshly collected tissue stored at −80 °C were used for western blot and qRT-PCR analyses. This study was approved by the Medical Research Ethics Committee of the Second Affiliated Hospital of Nanchang University, and all patients provided their written consent.

### Cell lines and culture

Human PC cell lines AsPC-1 (TCHu 8), PANC-1 (SCSP-535), and SW1990 (TCHu201) were purchased from the National Collections of Authenticated Cell Cultures, Chinese Academy of Sciences. The human PC cell line BxPC-3 (CL-0042) was purchased from Procell (Wuhan, China). The HPDE6-C7 cell line was maintained in our lab. The above cell lines were verified by short tandem repeat sequence identification of the cell bank, and all of them were free of mycoplasma contamination. PANC-1 and HPDE6-C7 cells were cultured in DMEM (Gibco, 11960–044) with 10% FBS (GIBCO) and 1% P/S (Solarbio, P1400), and AsPC-1 and BxPC-3 cells were cultured in RPMI-1640 (GIBCO, 31800022) with 10% FBS (GIBCO) and 1% P/S (Solarbio, P1400) in a 95% air, 5% CO_2_ incubator at 37 °C. SW 1990 cells were cultured in Leibovitz’s L-15 (PM151010) with 10% FBS (164210–500) and 1% P/S (PB180120) in a 100% air 37 °C incubator.

GEM-resistant PC cell lines (PANC-1/GR, AsPC-1/GR, and BxPC-3/GR) were established in our lab as described previously [65]. Briefly, cells were exposed to GEM at an initial concentration of 1/5th of the parent cell IC50 and cultured for 48 h. The culture medium was then discarded, the cells were washed twice with PBS, and the drug-free medium was replaced with normal culture medium. When the cells returned to normal growth, the above process was repeated eight times. After the cells grew steadily at this concentration, the drug concentration was increased, and the culturing was continued. The concentration of GEM was gradually increased until the resistance index (RI) > 5 (RI = IC50_drug-resistant cell_ /IC50_parental cell_), which lasted approximately 10 months. To induce EMT, we added 10 ng/mL of transforming growth factor-β (TGF-β) to the culture medium for 48 h after the normal culture of drug-resistant cell lines for 24 h [66].

### Reagents and antibodies

GEM and CHX were purchased from Selleck, puromycin dihydrochloride and MG-132 were purchased from Sigma, and TGF-β was purchased from R&D Systems. Antibodies and dilution ratios were as follows: anti-FAT10 (Boster, BM4765; 1:300), anti-FOXM1 (Santa Cruz, sc-376471; 1:300), anti-GAPDH (Proteintech, 60004-1-Ig; 1:10,000), anti-E-cadherin (Proteintech, 20874-1-AP; 1:10000), anti-N-cadherin (Proteintech, 22018-1-AP; 1:5000), anti-Vimentin (Proteintech, 60330-1-Ig; 1:10000), anti-Maspin(Proteintech, 11722-1-AP; 1:200), Goat anti-Rabbit IgG (H + L) Cross-Adsorbed Secondary Antibody, Alexa Fluor 546 (Invitrogen, A-11010; 1:1000), Goat anti-mouse IgG (H + L) cross-adsorbed secondary antibody, Alexa Fluor 488 (Invitrogen, A-11001; 1: 1000).

### Plasmid and shRNA transfection

The shRNA-mediated RNA duplex of FAT10 and FOXM1 was synthesized by GenePharma (Shanghai, China). FAT10, FAT10ΔGG, and FOXM1 plasmids were constructed and obtained from Focus Biotechnology (Nanchang, China). The shRNA and plasmid were transfected into PC cells using Lipofectamine 3000 transfection reagent (Invitrogen, L3000015). Lastly, puromycin was used to screen and establish PC cell lines stably transfected with sh-NC or sh-FAT10#2 plasmid. Table S2 lists the sequences of plasmid and shRNA used in the present study.

### RNA extraction and quantitative real-time PCR (qRT-PCR)

Total RNA was extracted from cultured PC cells and tissues using TRIzol reagent (Invitrogen, 15596026) according to the instructions of the manufacturer. Total RNA was quantified using an Evolution 350 spectrophotometer (Thermo). RNA reverse transcription was performed using the PrimeScript reverse RT reagent Kit with gDNA Eraser (TaKaRa, RR047A). Quantitative PCR was performed using TB Green ®Premix Ex Taq quantitative (Tli RNaseH Plus) (TaKaRa, RR420A). The gene expression level of each sample was normalized to the expression of GAPDH mRNA, and the 2^−ΔΔct^ method was used to calculate the gene expression level of each sample. Table S2 lists the sequences of all PCR primers used.

### Co-IP

Precooled RIPA lysate and PMSF (100:1) were used to lyse BxPC-3 and PANC-1 cells for 10 min, and cell lysates were centrifuged at 12,000 rpm for 5 min. The supernatant was incubated with 2 μg primary antibody for 1 h, after which 40 μL protein A/G PLUS-Agarose (Santa Cruz, sc-2003) was added, and the plate was incubated for 24 h on a shaker at 4 °C. After centrifugation at 2500 rpm for 5 min, the precipitate was collected. The pellet was washed with 1 mL prechilled RIPA lysis buffer four times. Finally, the precipitate was dissolved in 40 μL of 1× electrophoresis sample buffer and boiled for 2 min, followed by western blotting or liquid chromatography−tandem mass spectrometry (LC−MS/MS).

### Western blot

PC tissues and cells were collected, and RIPA lysis buffer was used to extract total protein. After determining the protein concentration by the BCA method, a protein loading buffer was added, and samples were boiled for 10 min. The proteins were electrophoresed using 10% or 7.5% SDS–PAGE and transferred to a 0.22 μm PVDF membrane. The membrane was blocked with 5% skim milk at room temperature for 1 h, incubated with the corresponding primary antibodies at 4 °C overnight, washed thrice for 10 min each with 1×TBST, and incubated with the secondary antibody of the corresponding species at room temperature for 1 h. After washing thrice with 1×TBST, an ECL reagent was used to expose and image the membranes. ImageJ software was used to analyze the data.

### LC-MS/MS analysis

Anti-FAT10 immunoprecipitates from PANC-1 cell lysates were extracted by the same co-IP assay described above. Electrophoresis sample buffer (1×, 20 μL) was used for electrophoresis. After electrophoresis, the SDS–PAGE gel was stained with Coomassie brilliant blue for 3 h and washed with the eluate overnight. The stained gel was used for LC-MS/MS analysis as previously described [9].

### IHC and immunofluorescence analyses

PC and corresponding adjacent tissues were fixed, paraffin-embedded, sectioned, deparaffinized, hydrated, subjected to antigen retrieval, and blocked with goat serum. Then, anti-FAT10 (Boster, BM4765; 1:100) and anti-Maspin (Proteintech, 11722-1-AP; 1:100) primary antibodies were added to the samples and incubated overnight at 4 °C. For IHC, the sections were labeled with an Envision HRP kit (DAKO) at room temperature for 30 min. 3,3′-Diaminobenzidine (DAKO) was added for color development at room temperature with incubation for 10 min, followed by counterstaining with Mayer’s hematoxylin (DAKO) for 2 min. For immunofluorescence analysis, the sections were incubated with corresponding fluorescent secondary antibodies for 30 min at room temperature, then DIPA was used to stain cell nuclei for 5 min. Finally, an anti-fluorescence quenched mounting medium was used to mount the slides.

### Cell viability assay

PC cells (1 × 10^4^ cells/100 μL) of each group in the logarithmic growth phase were inoculated into 96-well plates. After the cells adhered to the wall, medium containing different GEM concentrations was added to the culture for 48 h. Then, 10 μL of every 100 μL of Cell Counting Kit-8 (GLPBIo, GK10001) medium was added and incubated for 2 h, and the absorbance at 450 nm was measured using a microplate reader. The OD value (taking the average of three wells) was used to calculate the cell survival rate, and the cell viability rate (%) = [(OD_Treatment_ − OD_Blank_)/(OD_control_ − OD_Blank_)] × 100%.

### Immunofluorescence

PC cells (3 × 10^4^ cells/mL) with different treatments were inoculated into 24-well plates and cultured for 24 h. Subsequently, the cells were fixed in 4% paraformaldehyde for 15 min and washed with PBS thrice for 3 min each. The cells were permeabilized using 0.5% Triton X-100 for 20 min at room temperature. After permeabilization, PBS was used to wash the cells again three times, and the PBS was then removed using absorbent paper. Next, 500 μL of 5% goat serum was added dropwise to each well to block non-specific binding for 30 min at room temperature. Anti-E-cadherin (Proteintech, 20874-1-AP; 1:200) and anti-Vimentin (Proteintech, 60330-1-Ig; 1:200) antibodies were added and incubated at 4 °C overnight. The cells were rinsed thrice with PBST and incubated with fluorescent secondary antibody for 1 h. Then, PBST was used for rinsing thrice, and DAPI was added to stain the nucleus for 5 min. After rinsing, the fluorescence intensity of cells in the treatment and control groups was observed under a fluorescence microscope.

### 5-Ethynyl-20-deoxyuridine (EdU) incorporation assay

Cells (3 × 10^4^ cells/100 μL) with different treatments were inoculated into 96-well plates and cultured for 24 h. The Cell-Light EdU Apollo488 In Vitro Kit (RiboBio, C10310–3) was used for the assay following the kit instructions [67]. Cell proliferation in the treatment and control groups was observed under a fluorescence microscope.

### Acridine orange/ethidium bromide (AO/EB) staining

Cells (1.5 × 10^4^ cells/100 μL) with different treatments were inoculated into 96-well plates and cultured for 24 h. AO/EB staining (Bestbio, Shanghai, China) was performed according to the manufacturer’s instructions. Briefly, the cells were incubated with AO/EB solution in the dark for 15 min, and the morphology of living cells and apoptotic cells in the different groups was immediately observed under a fluorescence microscope. Images were acquired, and the percentage of apoptotic cells was evaluated using ImageJ software.

### Subcutaneous xenograft experiments

A subcutaneous xenograft mouse model was used to evaluate the tumor-forming ability of PANC-1 cells with stable knockdown of FAT10 with or without GEM treatment. Male BALB/c nude mice that were 4 weeks old (18–22 g) were purchased from Hangzhou Ziyuan Experimental Animal Technology Co., Ltd. PANC-1 cells (1 × 10^7^) stably transfected with sh-NC and shFAT10 were resuspended in 200 μL PBS and subcutaneously injected into the backs of nude mice. One week later, mice in the experimental group were treated with an intraperitoneal injection of GEM (50 mg/kg) twice a week. The length and width of the tumors were measured using calipers every 5 days, and the tumor volume (V) was calculated as follows: V = 0.52 × length × width^2^. Experimental nude mice were euthanized at the end of the observation period, and then the tumors were removed and imaged. All animal experiments were approved by the Laboratory Animal Science Center of Nanchang University.

### Statistical analysis

All data are expressed as mean ± standard error of mean by using GraphPad Prism 8.0 (GraphPad, La Jolla, CA, USA). We used the Wilcoxon rank-sum test to determine the differences in the expression of *FAT10* in PC and neighboring noncancerous tissues. Kaplan–Meier survival curves were analyzed using the Log-rank test. Inhibition curves were fitted by nonlinear regression, and GEM IC50s were calculated using GraphPad Prism 8 software. Spearman correlation was used to analyze the correlation between *FAT10* and EMT pathway score. Student’s t-tests and two-tailed distributions were used to compute significant differences. All experiments were independently repeated thrice. Differences were considered to indicate statistical significance at *p* < 0.05.

## Supplementary information

Supplementary Figure legends

Supplementary Figure 1

Supplementary Figure 2

Supplementary Figure 3

Supplementary Table 1

Supplementary Table 2.

author contribution

checklist

Original Data File

## Data Availability

All data generated or analyzed during this study are included in this published article. Additional datasets used and/or analyzed during the current study are available from the corresponding author on reasonable request.
