# Supplementary material for: FAT10 promotes chemotherapeutic resistance in pancreatic cancer by inducing epithelial-mesenchymal transition via stabilization of FOXM1 expression
Source: Cell Death Dis. 2022 May 25;13(5):497. doi: 10.1038/s41419-022-04960-0 (PMC9132907; doi:10.1038/s41419-022-04960-0)
Supplement: Supplementary file 1 — Supplementary Figure legends [file 41419_2022_4960_MOESM1_ESM.docx]

**SUPPLEMENTAL FIGURE LEGENDS**

**Supplementary Figure 1. Efficiency of FAT10 interference or overexpression in PC cells.** (**A-B**) Protein and mRNA levels of FAT10 were assessed in PANC-1 cells transfected with sh-NC or sh-FAT10 by qRT-PCR and western blotting. GAPDH was used as a loading control. (**C-D**) Protein and mRNA levels of FAT10 were assessed in AsPC-1 cells transfected with vector or HA-FAT10 by qRT-PCR and western blotting. GAPDH was used as a loading control. Data represent the mean ± SD of triplicate experiments and were statistically analyzed using Student’s t-test, **p*<0.05, ***p*<0.01, ****p*<0.001.

**Supplementary Figure 2. Overexpression of FAT10 increases the resistance of PC to GEM chemotherapy. (A-B)** EdU staining was used to detect the effect of overexpression of

FAT10 on the proliferation rate of PC cells treated with GEM. **(C-D)** AO/EB staining was used to detect the apoptotic rate of PC cells overexpressing FAT10 when treated with GEM. Data represent the mean ± SD of triplicate experiments and were statistically analyzed by one-way ANOVA. **p*<0.05, ***p*<0.01, ****p*<0.001

**Supplementary Figure 3. Detection of mRNA levels of FAT10 and FOXM1.**

The mRNA levels of FAT10 and FOXM1 were assessed by qRT-PCR in PANC-1 cells transfected with vector or HA-FAT10. GAPDH was used as a loading control. Data represent the mean ± SD of triplicate experiments and were statistically analyzed by Student’s t-test. ^**^*p*<0.01, NS, *p*>0.05.

**Supplementary Table 1. Identification of FAT10 interacting proteins by mass spectrometry.**

**Supplementary Table 2. Primers, plasmids and shRNA target sequences.**
