# Supplementary figures and images for: FAT10 promotes chemotherapeutic resistance in pancreatic cancer by inducing epithelial-mesenchymal transition via stabilization of FOXM1 expression
Source: Cell Death Dis. 2022 May 25;13(5):497. doi: 10.1038/s41419-022-04960-0 (PMC9132907; doi:10.1038/s41419-022-04960-0)

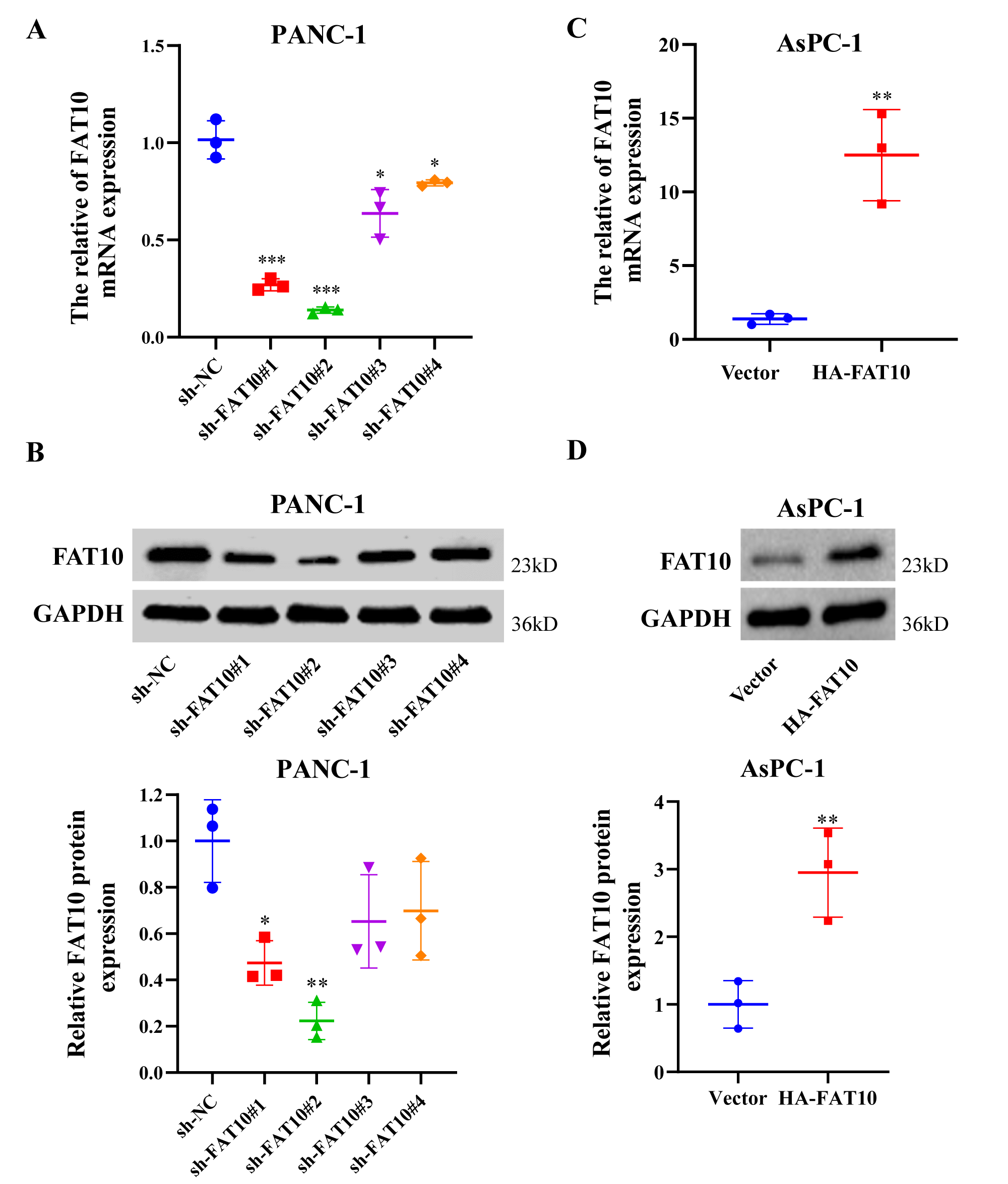

Supplement: Supplementary file 2 — Supplementary Figure 1 [file 41419_2022_4960_MOESM2_ESM.tif]

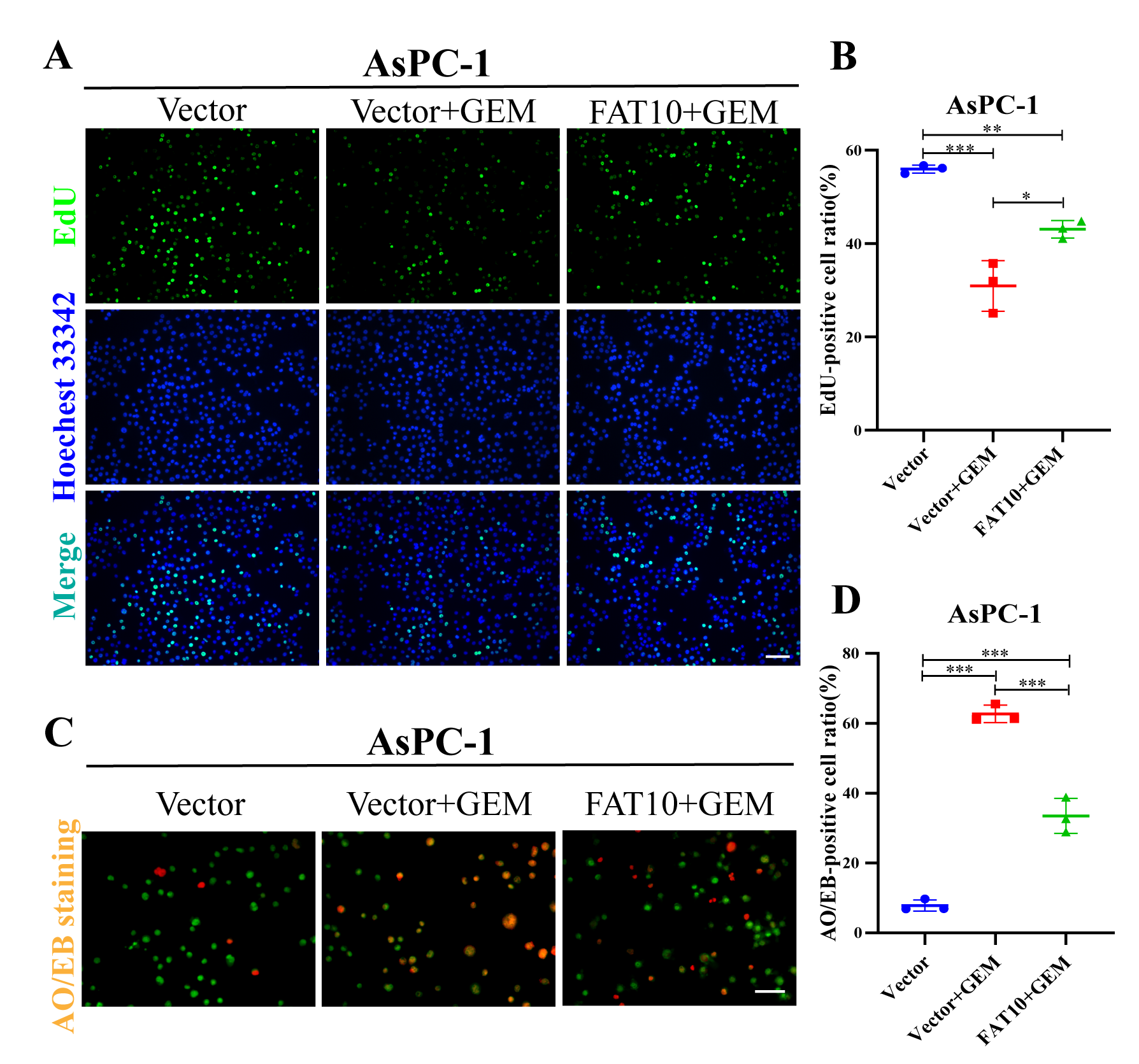

Supplement: Supplementary file 3 — Supplementary Figure 2 [file 41419_2022_4960_MOESM3_ESM.tif]

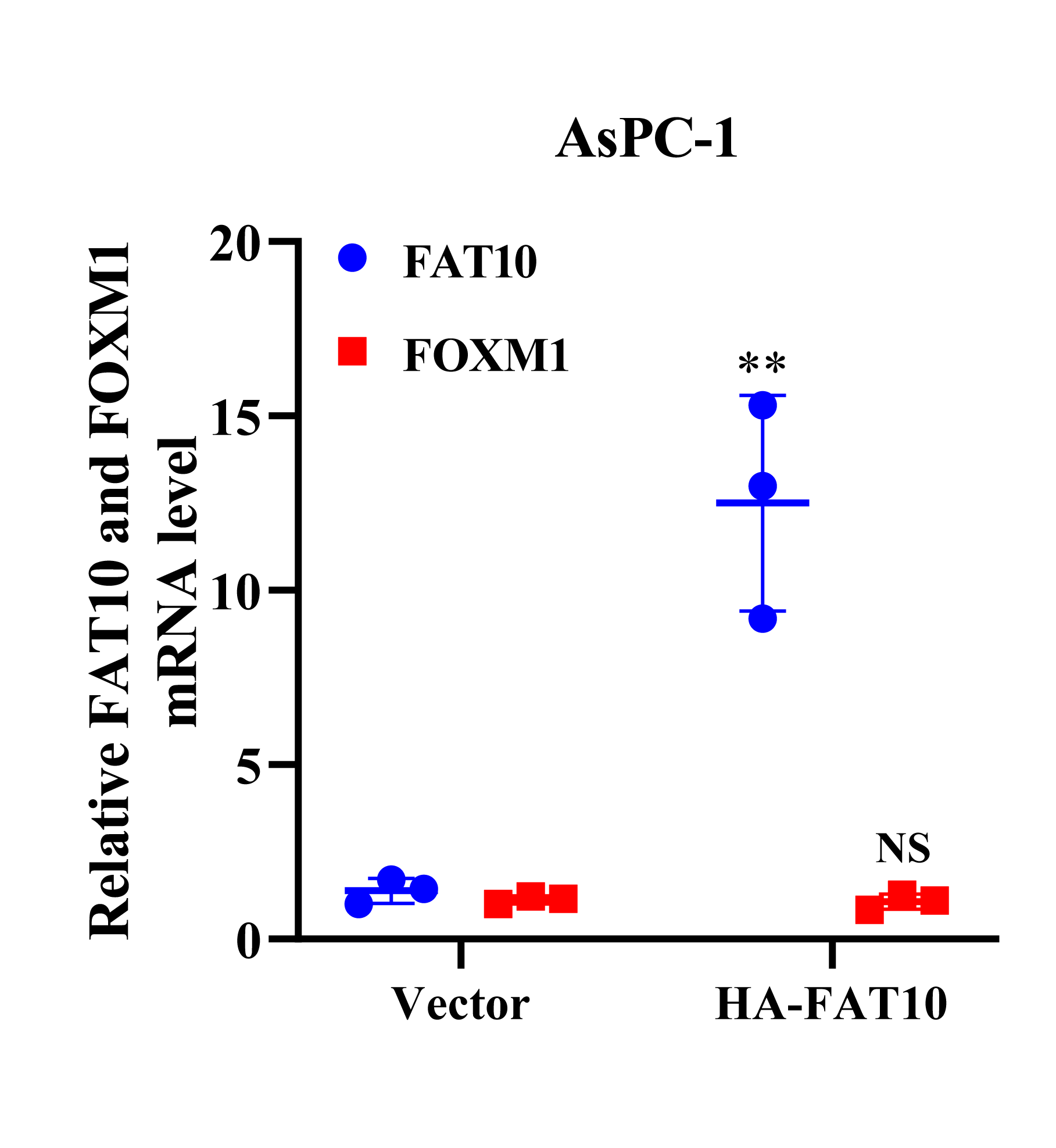

Supplement: Supplementary file 4 — Supplementary Figure 3 [file 41419_2022_4960_MOESM4_ESM.tif]
