# Supplementary material for: FAT10 promotes chemotherapeutic resistance in pancreatic cancer by inducing epithelial-mesenchymal transition via stabilization of FOXM1 expression
Source: Cell Death Dis. 2022 May 25;13(5):497. doi: 10.1038/s41419-022-04960-0 (PMC9132907; doi:10.1038/s41419-022-04960-0)
Supplement: Supplementary file 7 — author contribution [file 41419_2022_4960_MOESM7_ESM.pdf]

DECLARATION OF CONTRIBUTIONS TO ARTICLE

ADMC

Manuscript Number: CDDIS-21-4595R

Journal Name: *Cell Death & Disease* (the 'Journal')

Proposed Title of the Contribution:

FAT10 promotes chemotherapeutic resistance in pancreatic cancer by inducing epithelial-mesenchymal transition via stabilization of FOXM1 expression (the 'Contribution)

Author(s):

Jinfeng Zhu, Jiefeng Zhao, Chen Luo, Zhengming Zhu, Xingyu Peng, Xiaojian Zhu, Kang Lin, Fanqin Bu, Wenjun Zhang, Qing Li, Kai Wang, Zhigang Hu, Xin Yu, Leifeng Chen, and Rongfa Yuan (the 'Authors')

For all *CDD* articles, each person named as an author in the published version must be able to show he or she has contributed substantially to the article.

Authorship credit should be based on 1) substantial contributions to conception and design, acquisition of data, or analysis and interpretation of data; 2) drafting the article or revising it critically for important intellectual content; and 3) final approval of the version to be published. Authors should meet conditions 1, 2 and 3.

Any person who cannot be shown to have made a substantial contribution to the article cannot be listed as an author in the final version. The name of any person who is deemed to have made a minor contribution can, however, appear in the Acknowledgments section of the article.

Please complete the table below to indicate the contributions of all named authors to the manuscript.

Author Full Name:	Specification of Contribution to the Manuscript:
Jinfeng Zhu	1) acquisition of data, analysis and interpretation of data; 2) drafting the article and 3) final approval of the version to be published.
Jiefeng Zhao	1) acquisition of data; 2) revising the article; and 3) final approval of the version to be published.
Chen Luo	1) analysis and interpretation of data; 2) revising the article; and 3) final approval of the version to be published.
Zhengming Zhu	1) conception and design; 2) revising the article critically for important intellectual content; and 3) final approval of the version to be published.
Xingyu Peng	1) acquisition of data; 2) drafting the article and 3) final approval of the version to be published.
Xiaojian Zhu	1) analysis and interpretation of data; 2) revising the article critically for important intellectual content; and 3) final approval of the version to be published.
Kang Lin	1) acquisition of data; 2) revising the article and 3) final approval of the version to be published.
Fanqin Bu	1) acquisition of data; 2) revising the article and 3) final approval of the version to be published.
Wenjun Zhang	1) acquisition of data; 2) revising the article; and 3) final approval of the version to be published.
Qing Li	1) acquisition of data; 2) revising the article; and 3) final approval of the version to be published.
Kai Wang	1) conception and design; 2) revising the article critically for important intellectual content; and 3) final approval of the version to be published.
Zhigang Hu	1) analysis and interpretation of data; 2) revising the article; and 3) final approval of the version to be published.
Xin Yu	1) analysis and interpretation of data; 2) revising the article; and 3) final approval of the version to be published.

Author Full Name:	Specification of Contribution to the Manuscript:
Leifeng Chen	1) conception and design; 2) revising the article critically for important intellectual content; and 3) final approval of the version to be published.
Rongfa Yuan	1) conception and design; 2) revising the article critically for important intellectual content; and 3) final approval of the version to be published.

Please complete the table below to indicate the contributions of all named authors to the figures.

Figure 1:

Zhu Jinfeng， Jiefeng Zhao and Qing li obtained the data, Luo Cheng analyzed the results, Peng Xingyu and Xiaojian Zhu explained the results， and Kang Lin sorted and combined the pictures;

Figure 2:

Zhu Jinfeng， Jiefeng Zhao and Kai Wang obtained the data, Luo Cheng and Zhigang Hu analyzed the results, Peng Xingyu， Xin Yu and Xiaojian Zhu explained the results， and Kang Lin sorted and combined the pictures;

Figure 3:

Xin Yu， Jiefeng Zhao and Kai Wang obtained the data, Luo Cheng and Zhigang Hu analyzed the results, Wenjun Zhang， Xin Yu and Xiaojian Zhu explained the results， and Kang Lin sorted and combined the pictures;

Figure 4:

Jinfeng Zhu， Leifeng Chen and Kai Wang obtained the data, Luo Cheng and Zhigang Hu analyzed the results, Peng Xingyu， Xin Yu and Xiaojian Zhu explained the results， and Kang Lin sorted and combined the pictures;

Figure 5:

Jiefeng Zhao， Fanqin Bu, Leifeng Chen and Kai Wang obtained the data, Luo Cheng and Jinfeng Zhu analyzed the results, Wenjun Zhang， Xin Yu and Xiaojian Zhu explained the results， and Kang Lin sorted and combined the pictures;

Figure 6:

Jiefeng Zhao, Fanqin Bu, Leifeng Chen and Kai Wang obtained the data, Luo Cheng and Jinfeng Zhu analyzed the results, Peng Xingyu, Wenjun Zhang and Xiaojian Zhu explained the results， and Kang Lin sorted and combined the pictures;

Figure7： Jiefeng Zhao, Leifeng Chen and Kai Wang obtained the data, Luo Cheng analyzed the results, Peng Xingyu， Xin Yu and Xiaojian Zhu explained the results， and Kang Lin sorted and combined the pictures;

Figure8： Leifeng Chen， Luo Cheng and Rongfa Yuan designed and plotted the mechanism diagram.

Signed for and on behalf of the Author(s):	Print Name:	Date:
Rongfa Yuan	Rongfa Yuan	April 28, 2022
