# Supplementary material for: FAT10 promotes chemotherapeutic resistance in pancreatic cancer by inducing epithelial-mesenchymal transition via stabilization of FOXM1 expression
Source: Cell Death Dis. 2022 May 25;13(5):497. doi: 10.1038/s41419-022-04960-0 (PMC9132907; doi:10.1038/s41419-022-04960-0)

## Slide 1
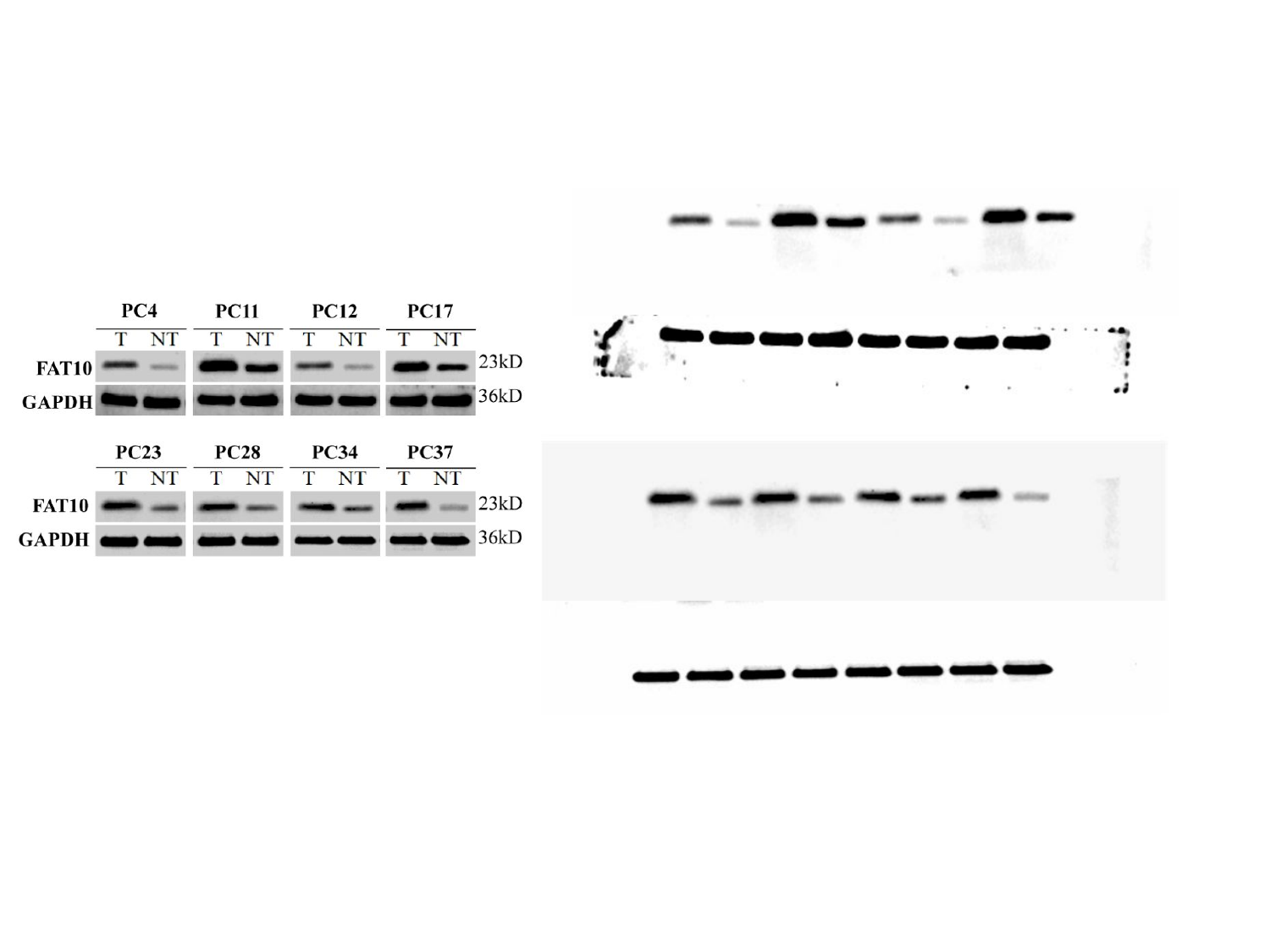

## Slide 2
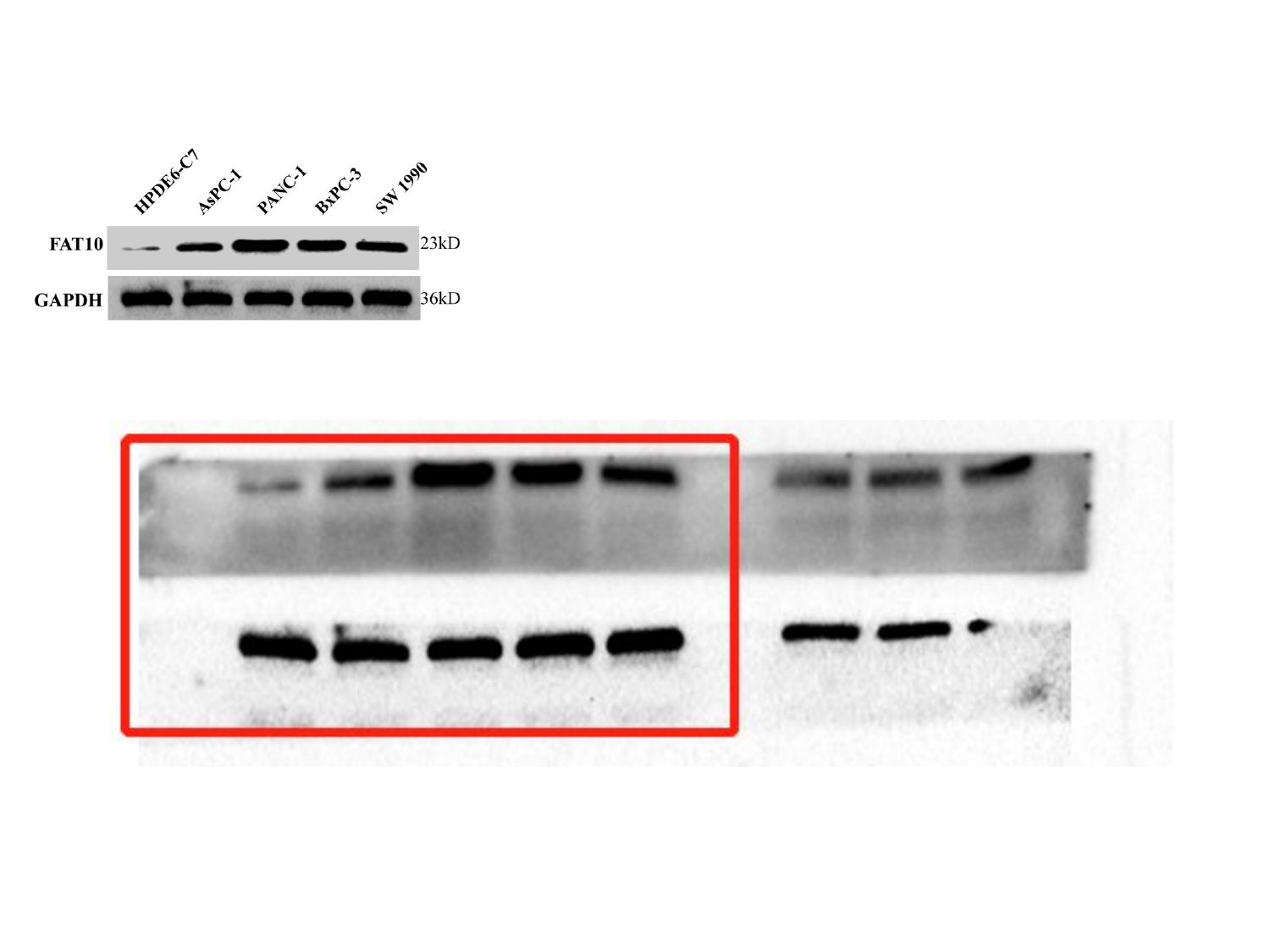

## Slide 3
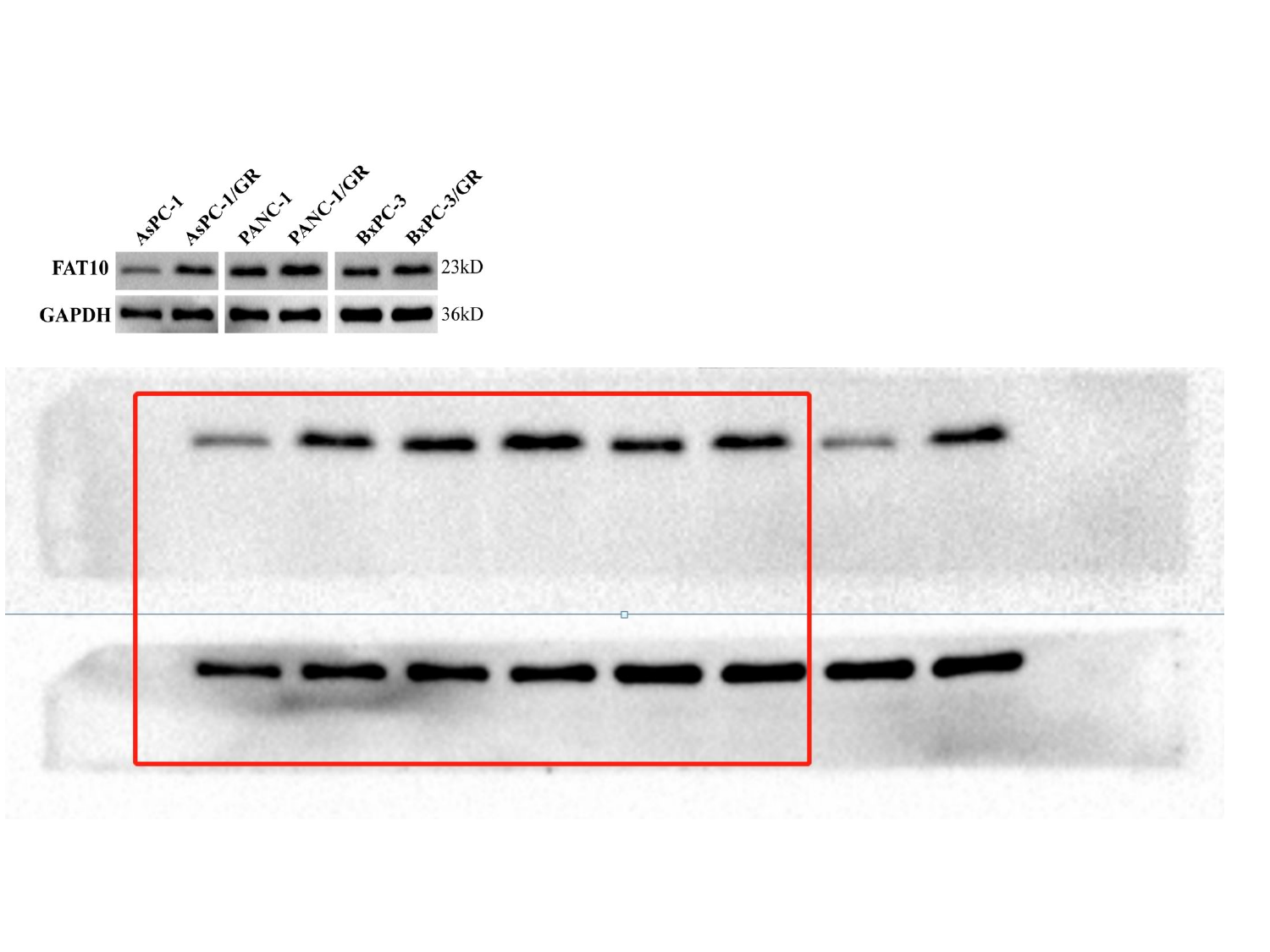

## Slide 4
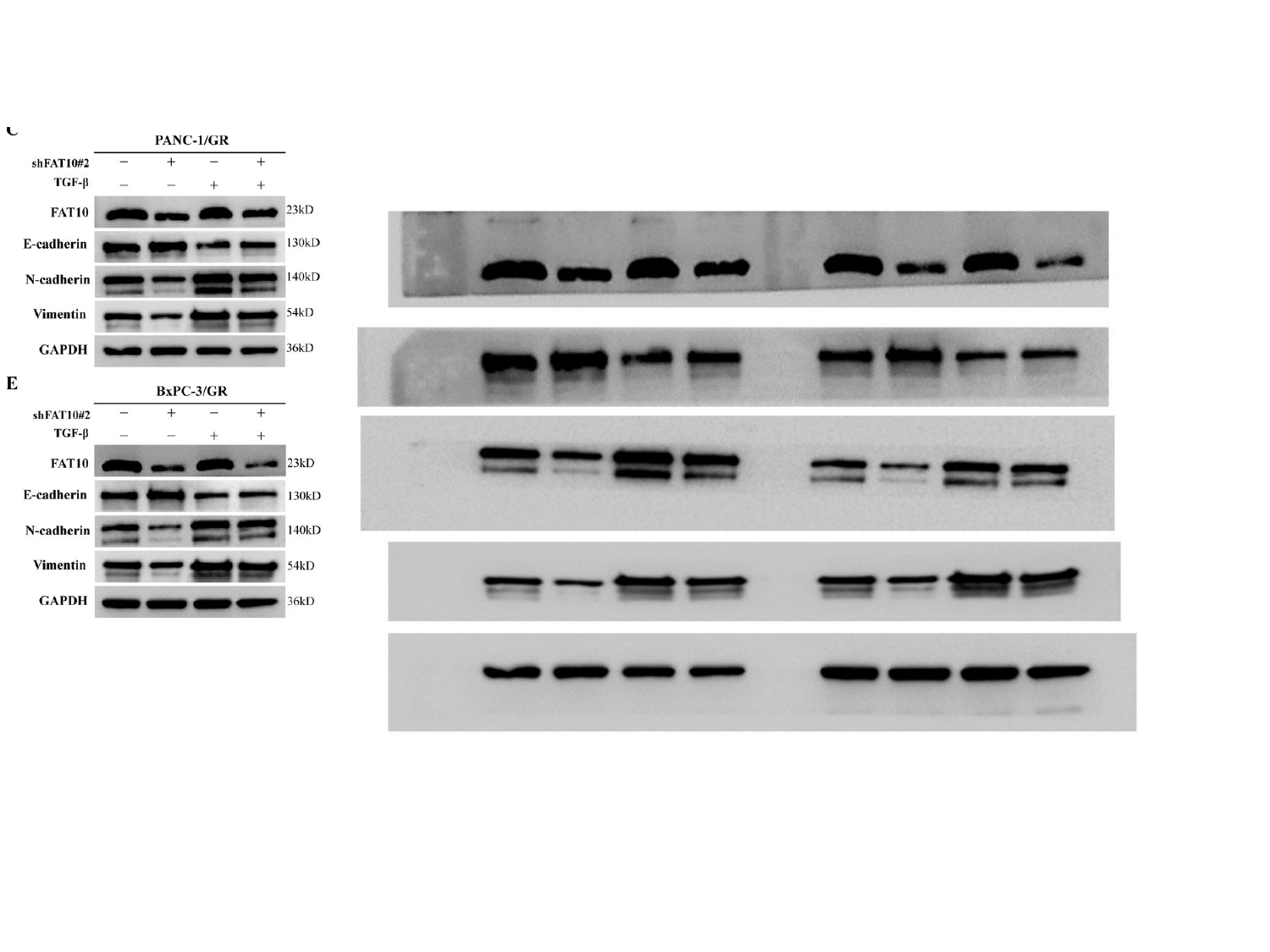

## Slide 5
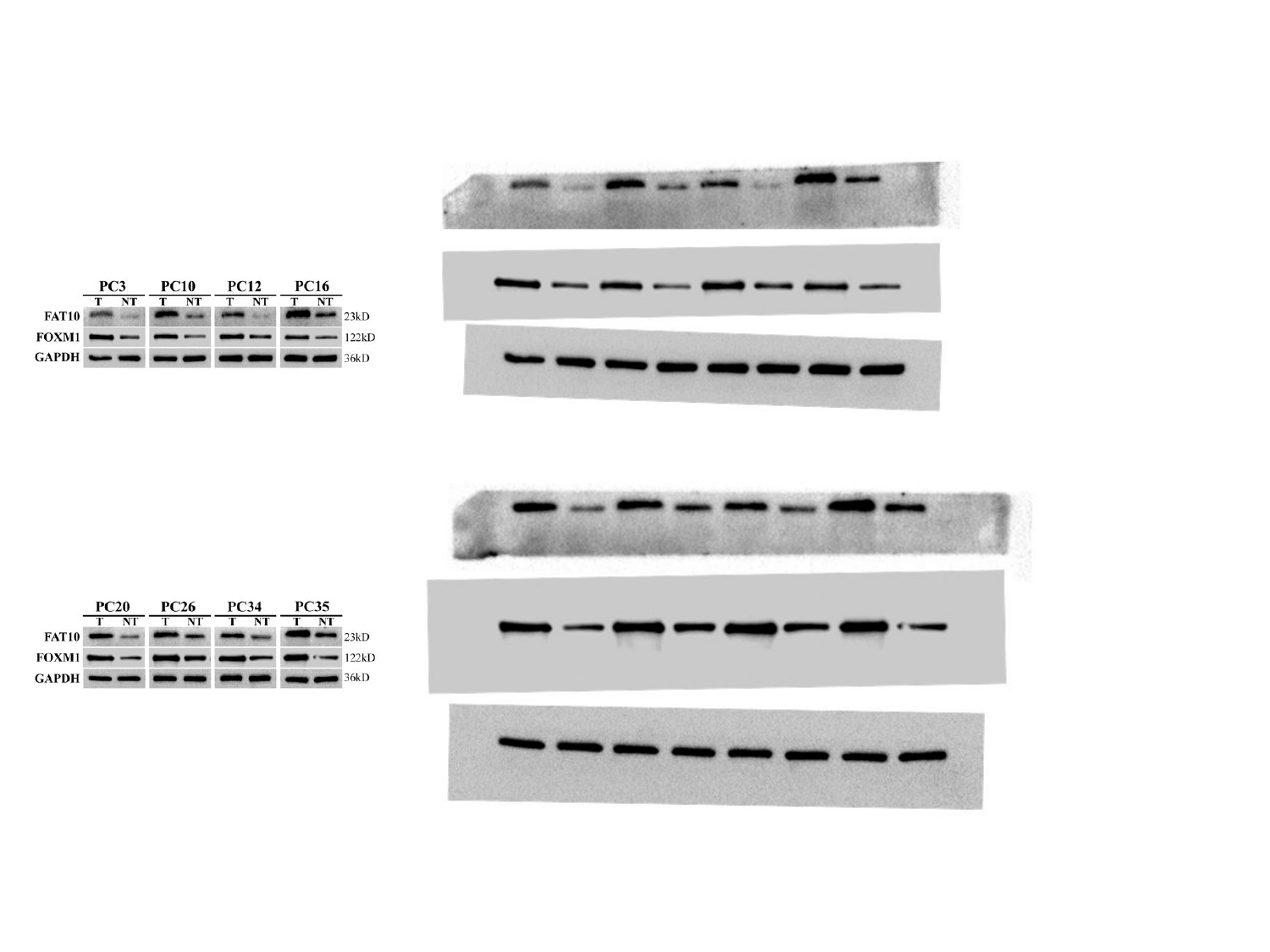

## Slide 6
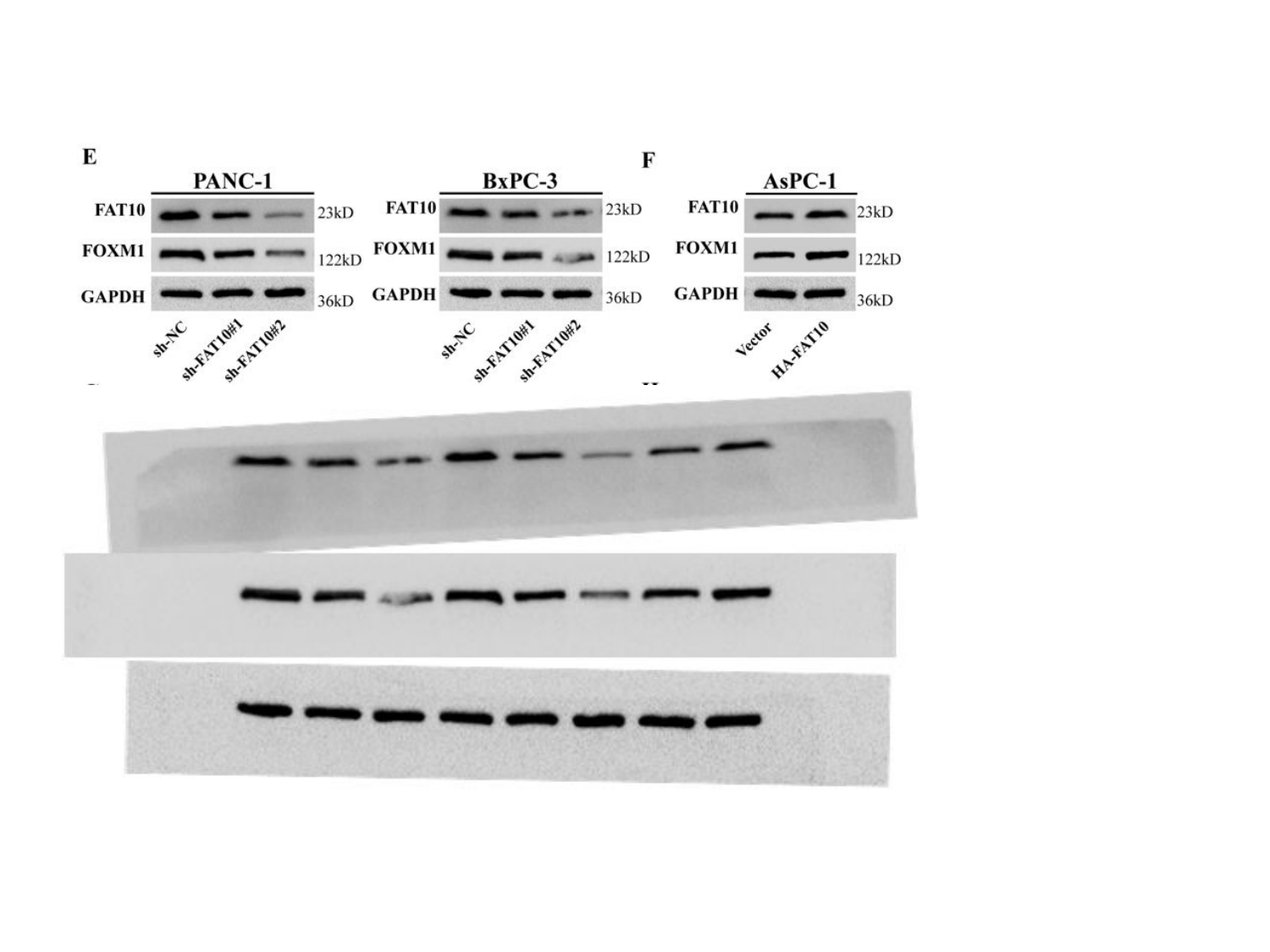

## Slide 7
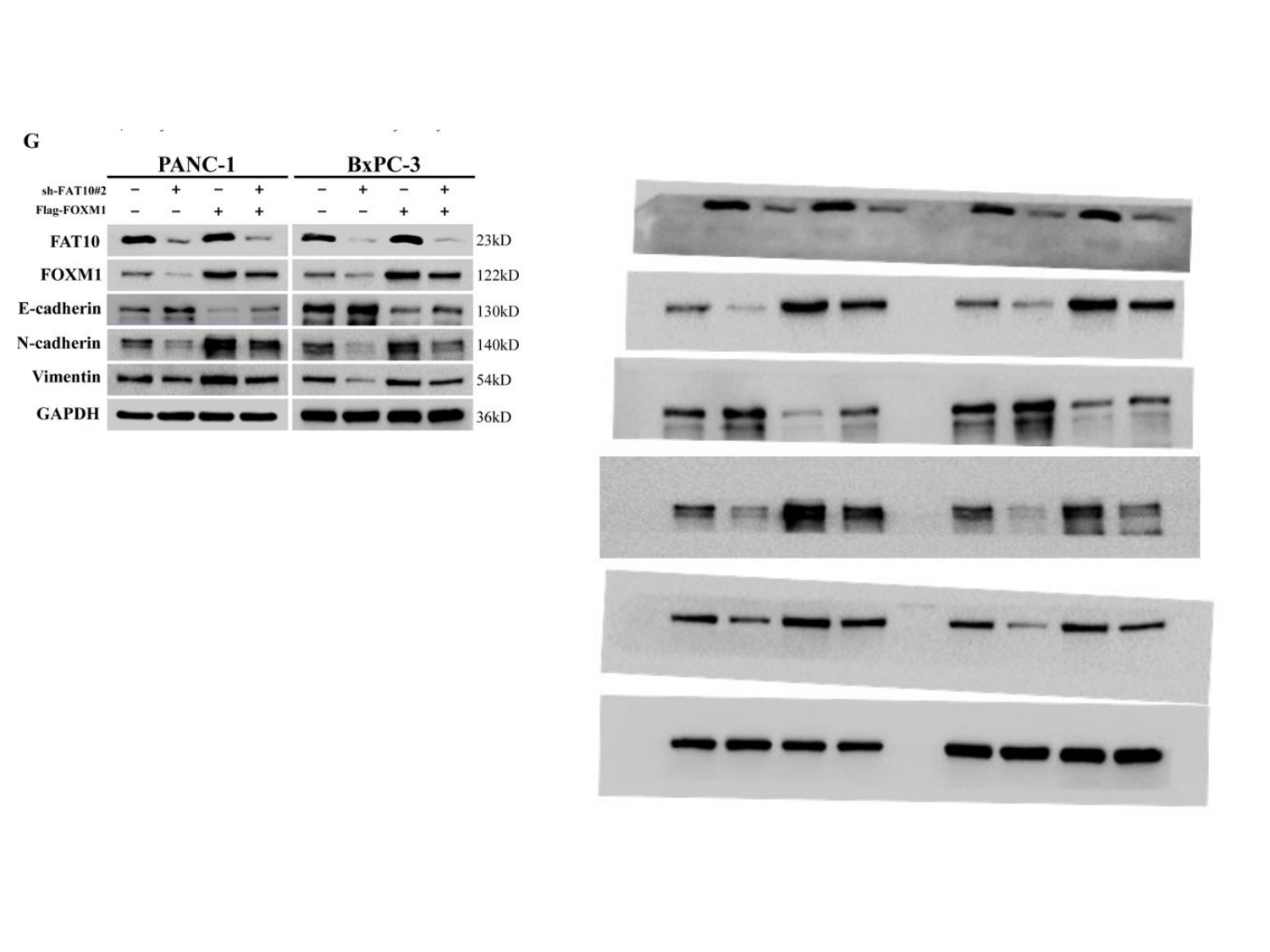

## Slide 8
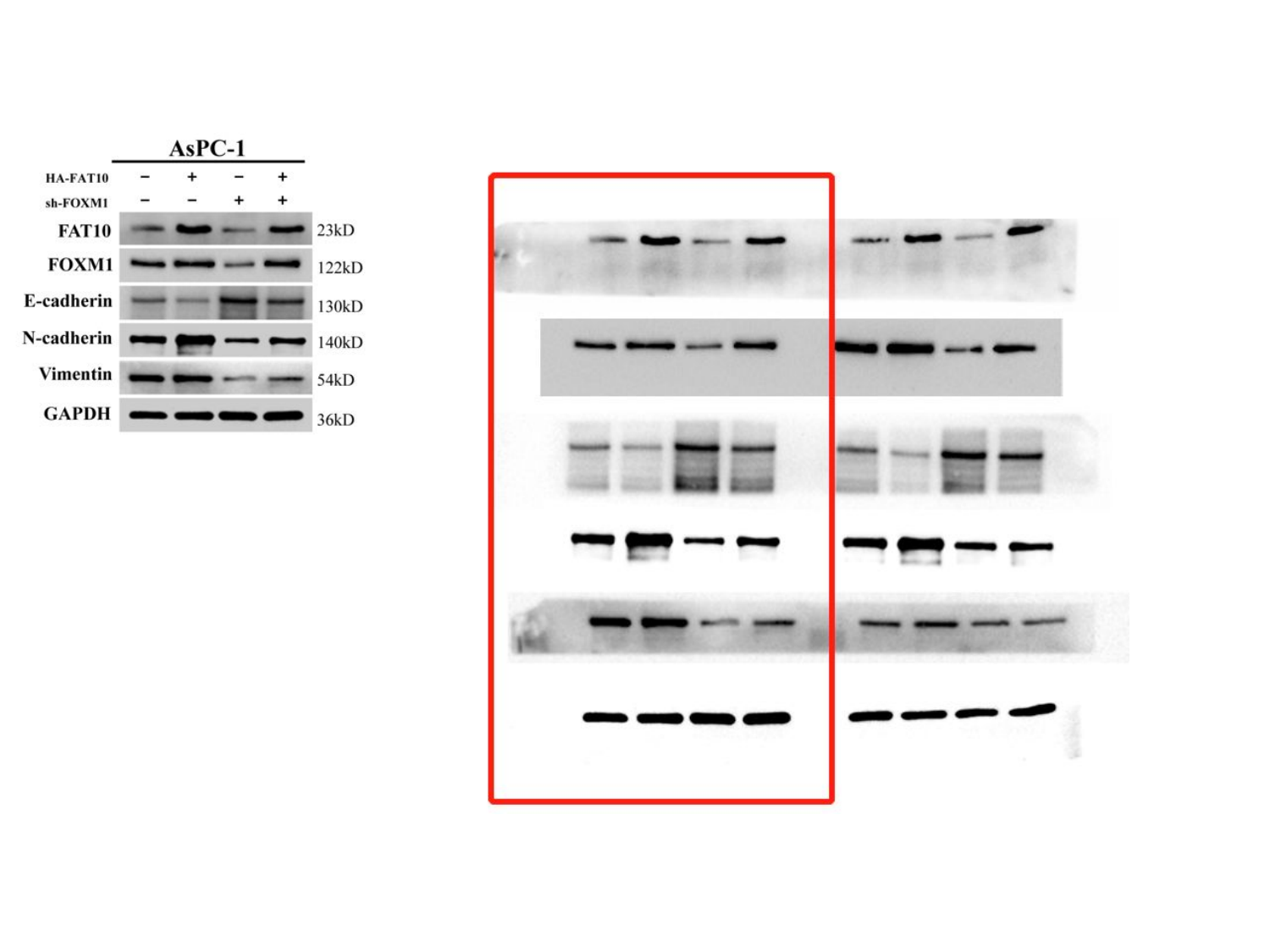

## Slide 9
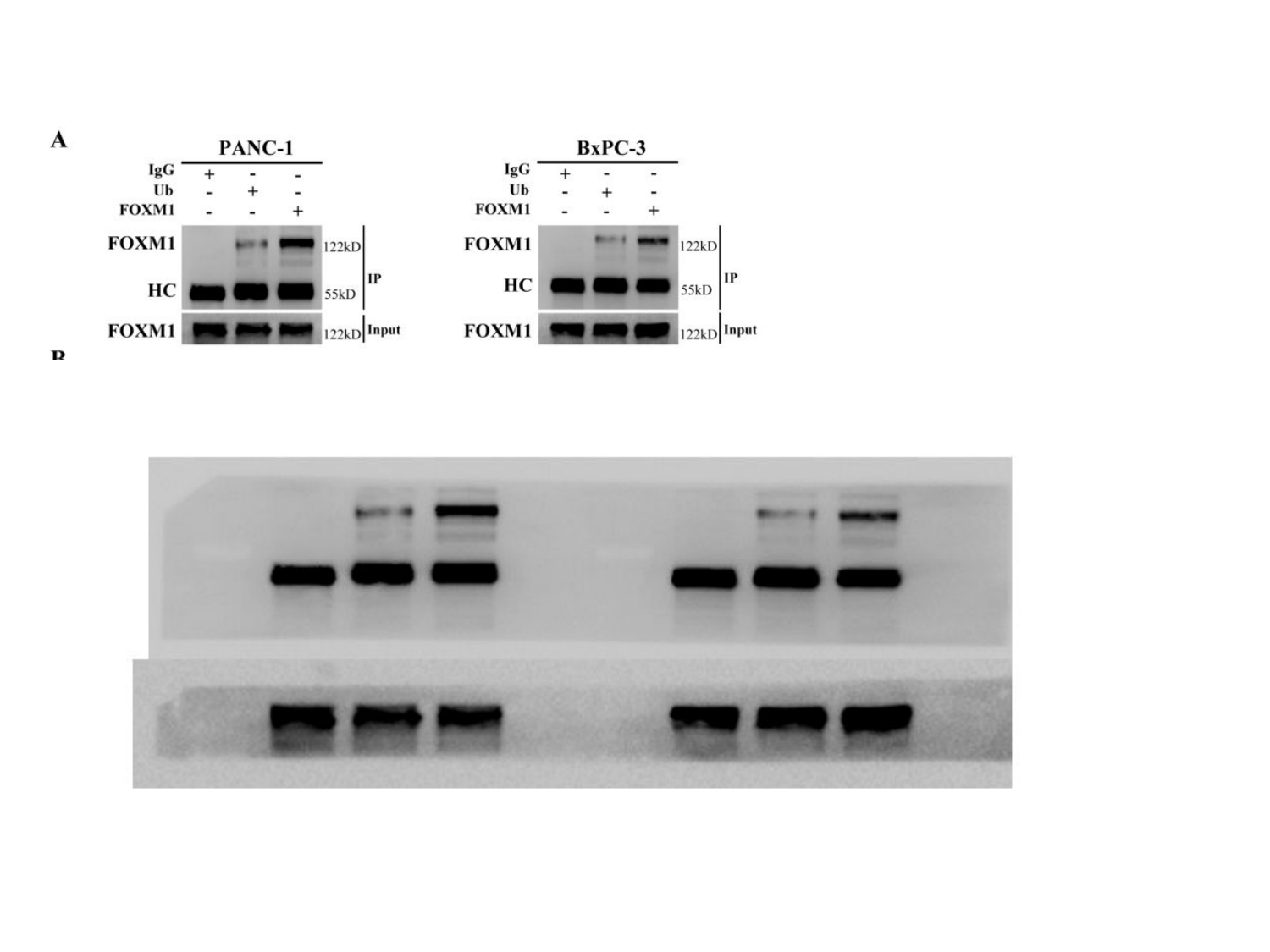

## Slide 10
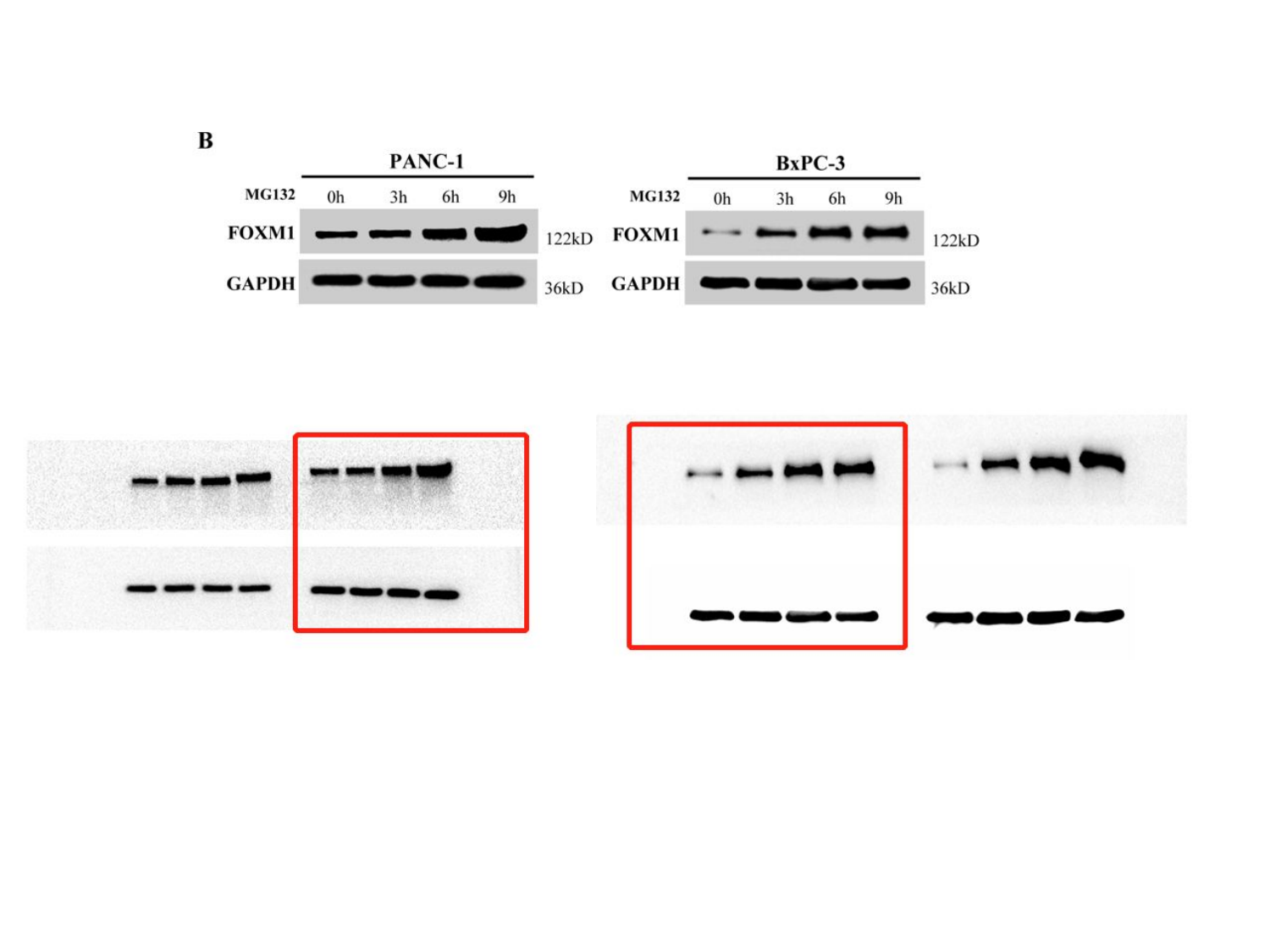

## Slide 11
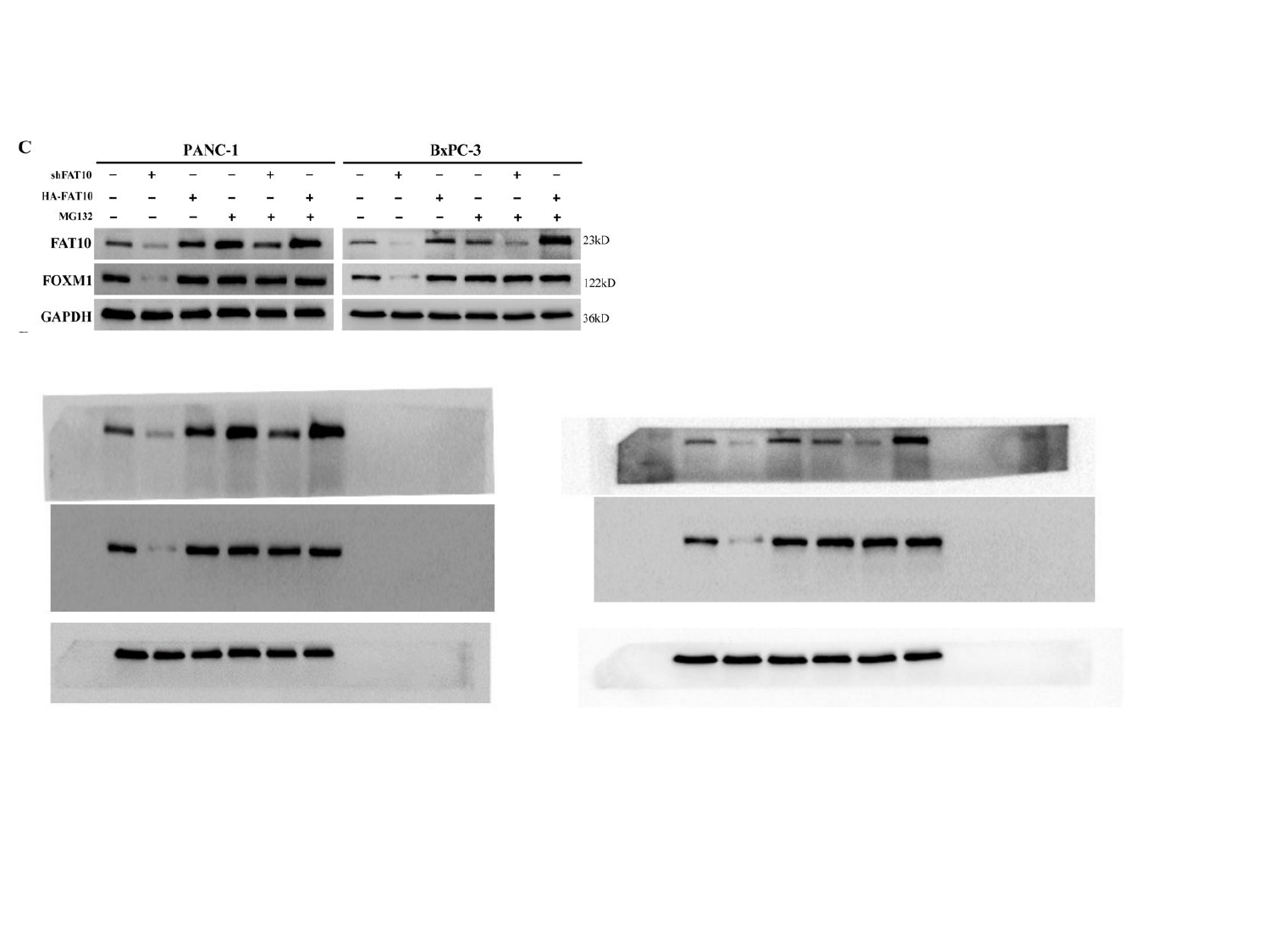

## Slide 12
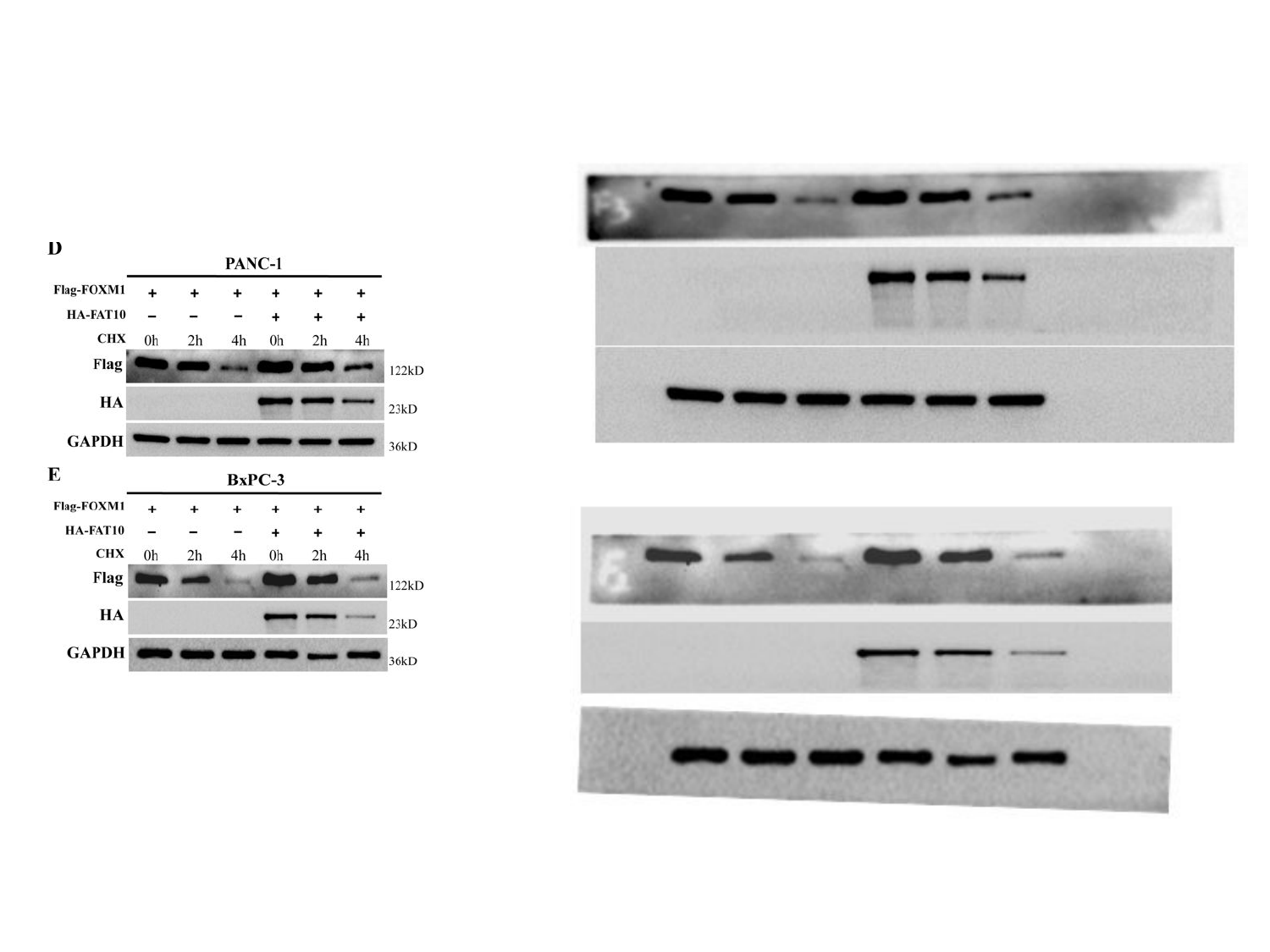

## Slide 13
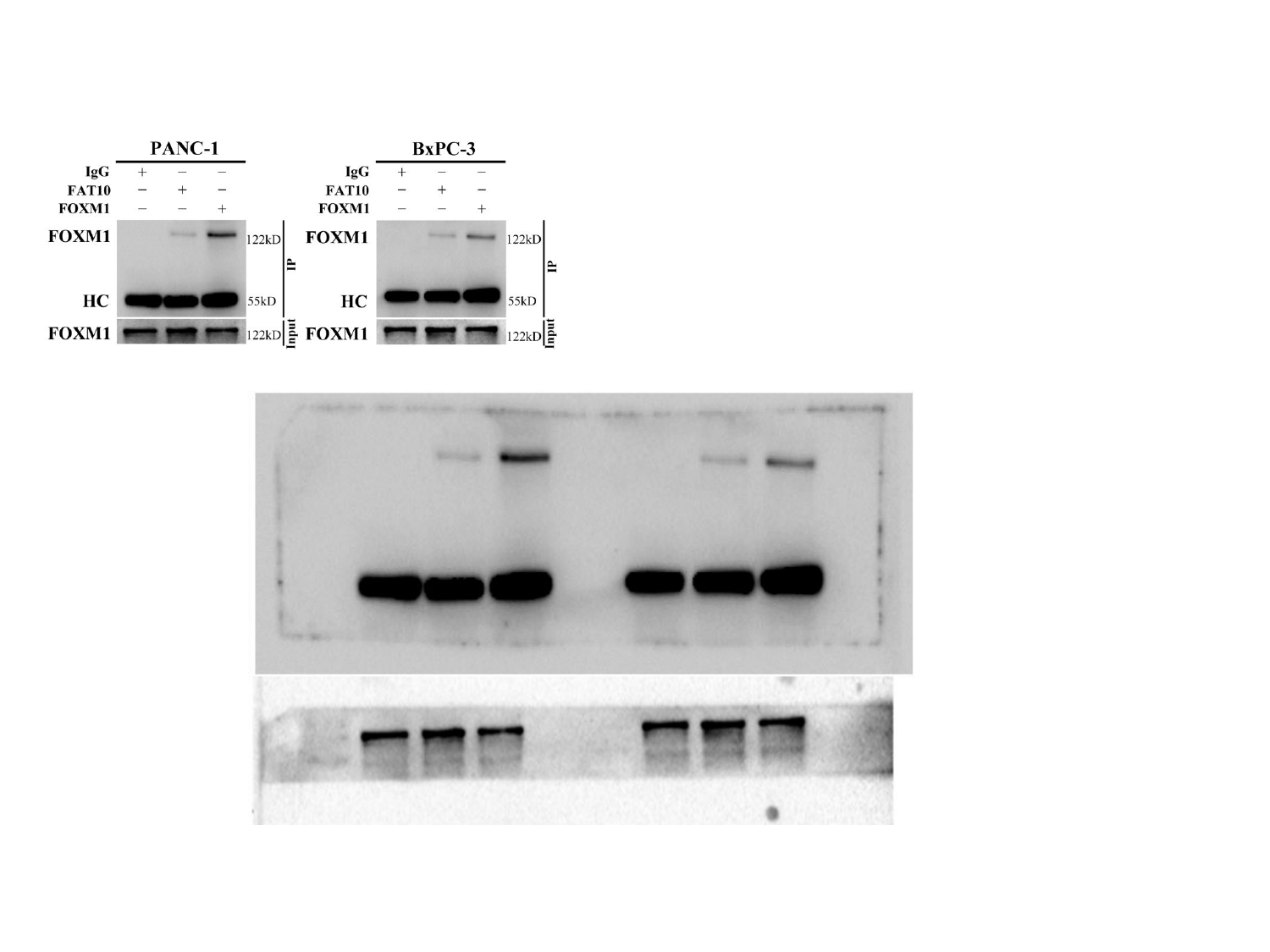

## Slide 14
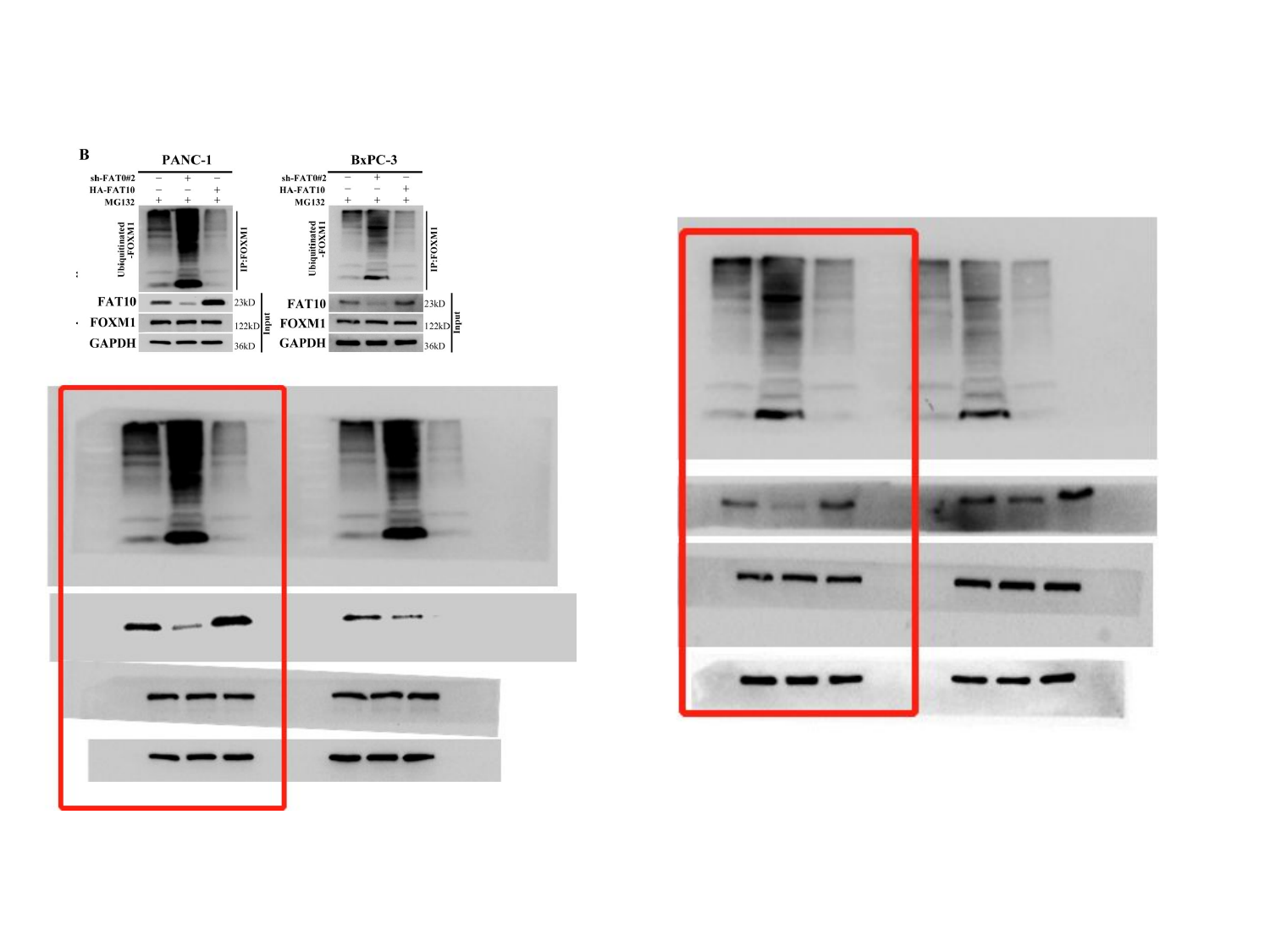

## Slide 15
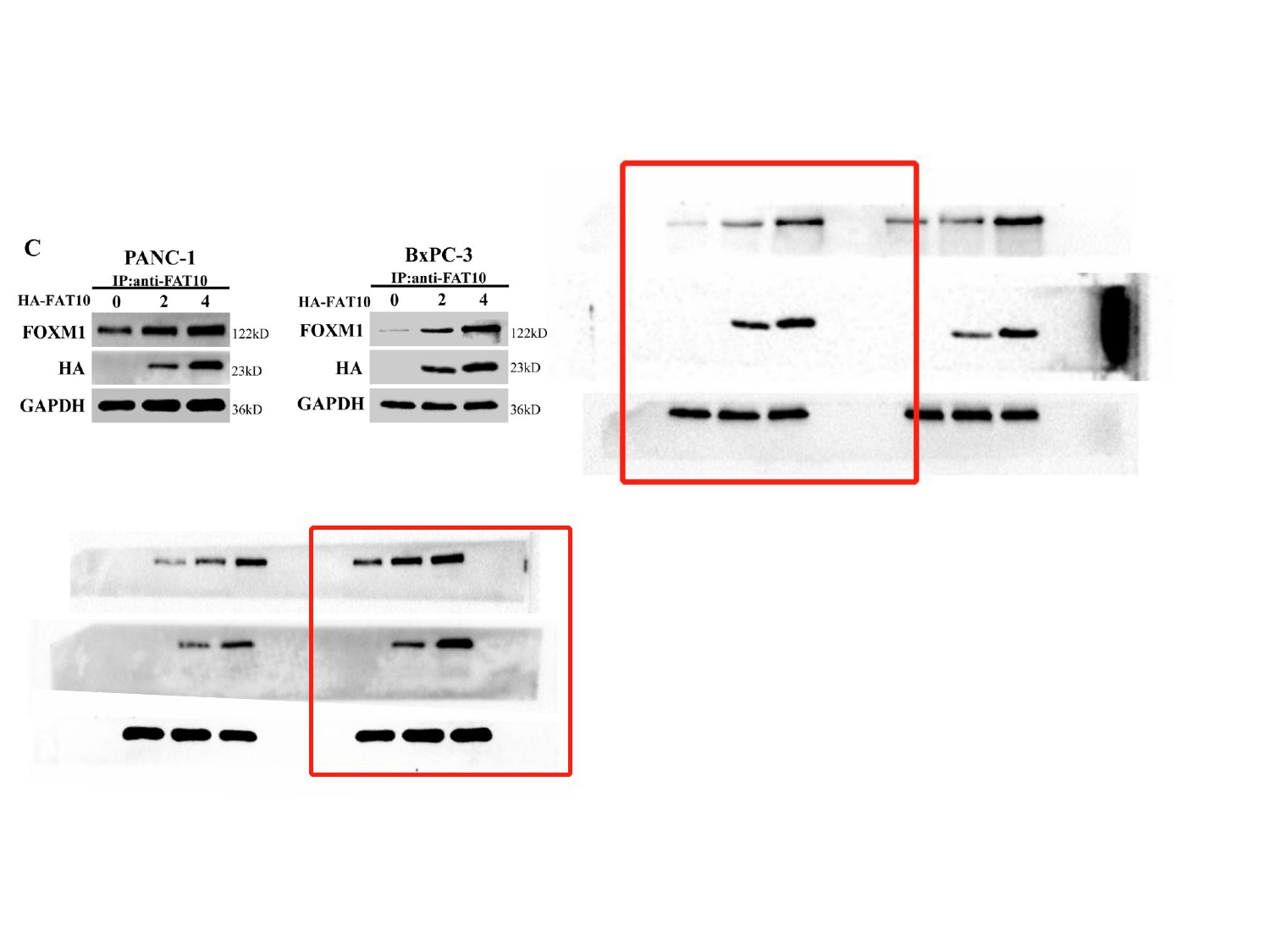

## Slide 16
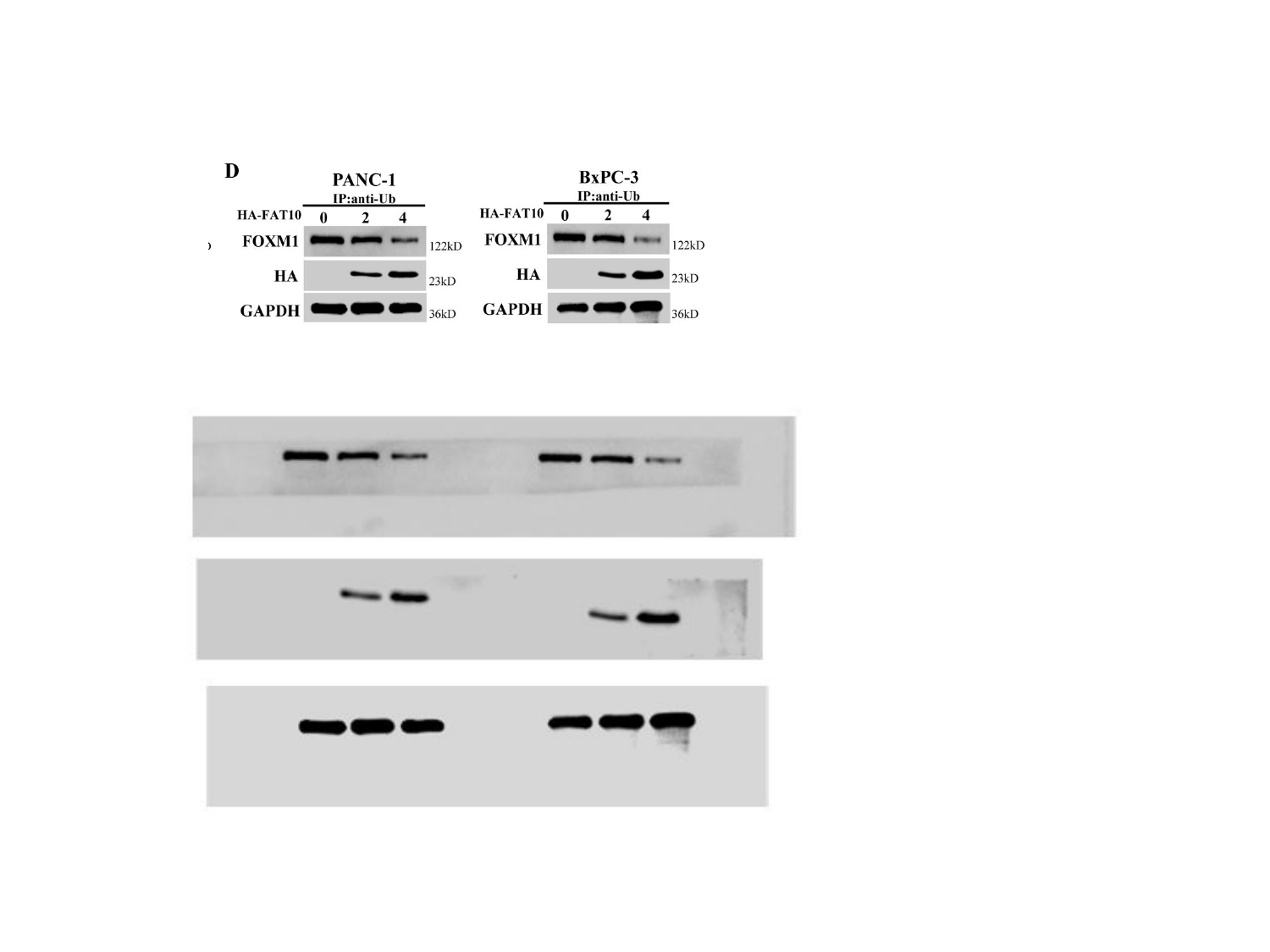

## Slide 17
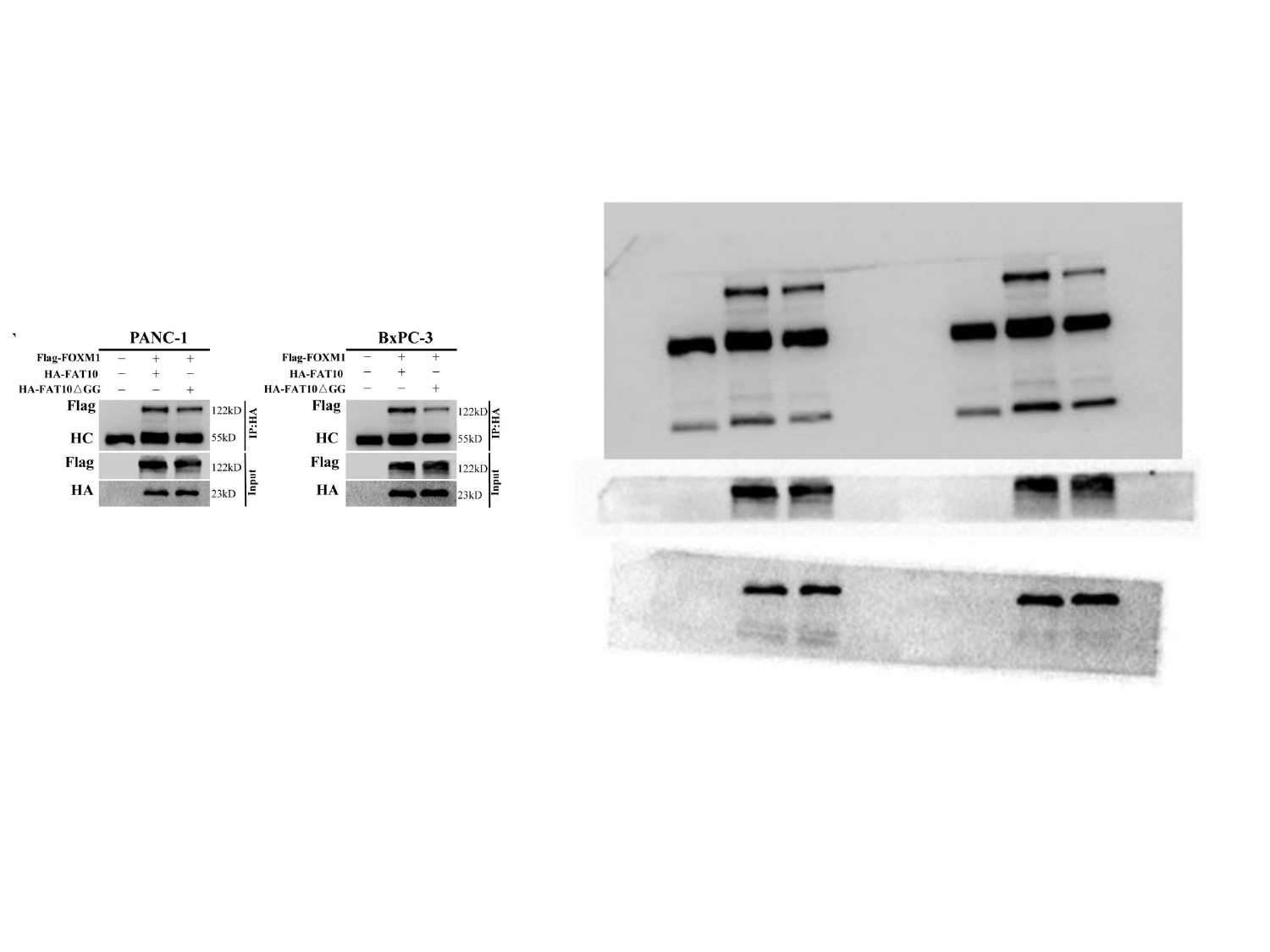

## Slide 18
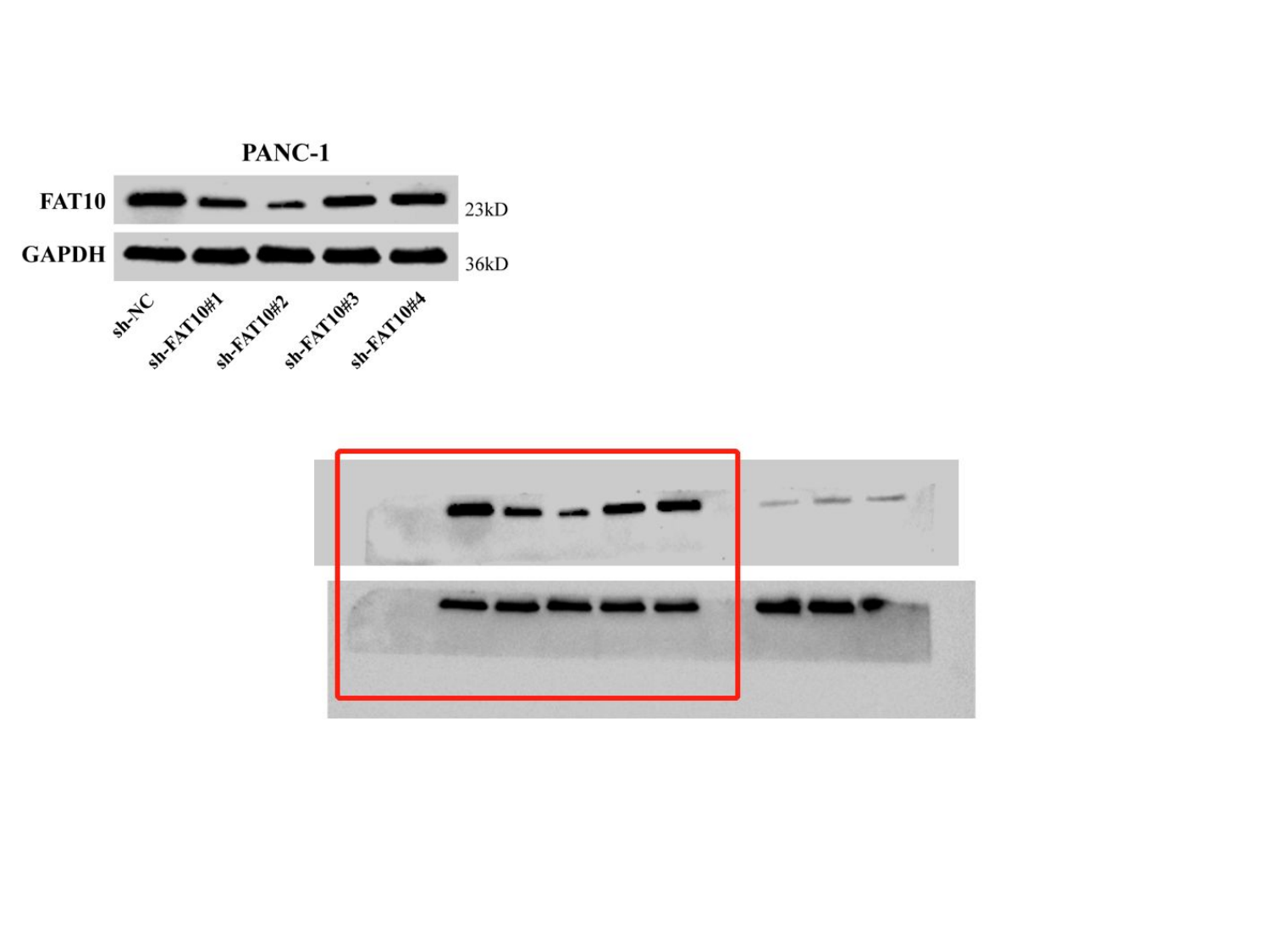

## Slide 19
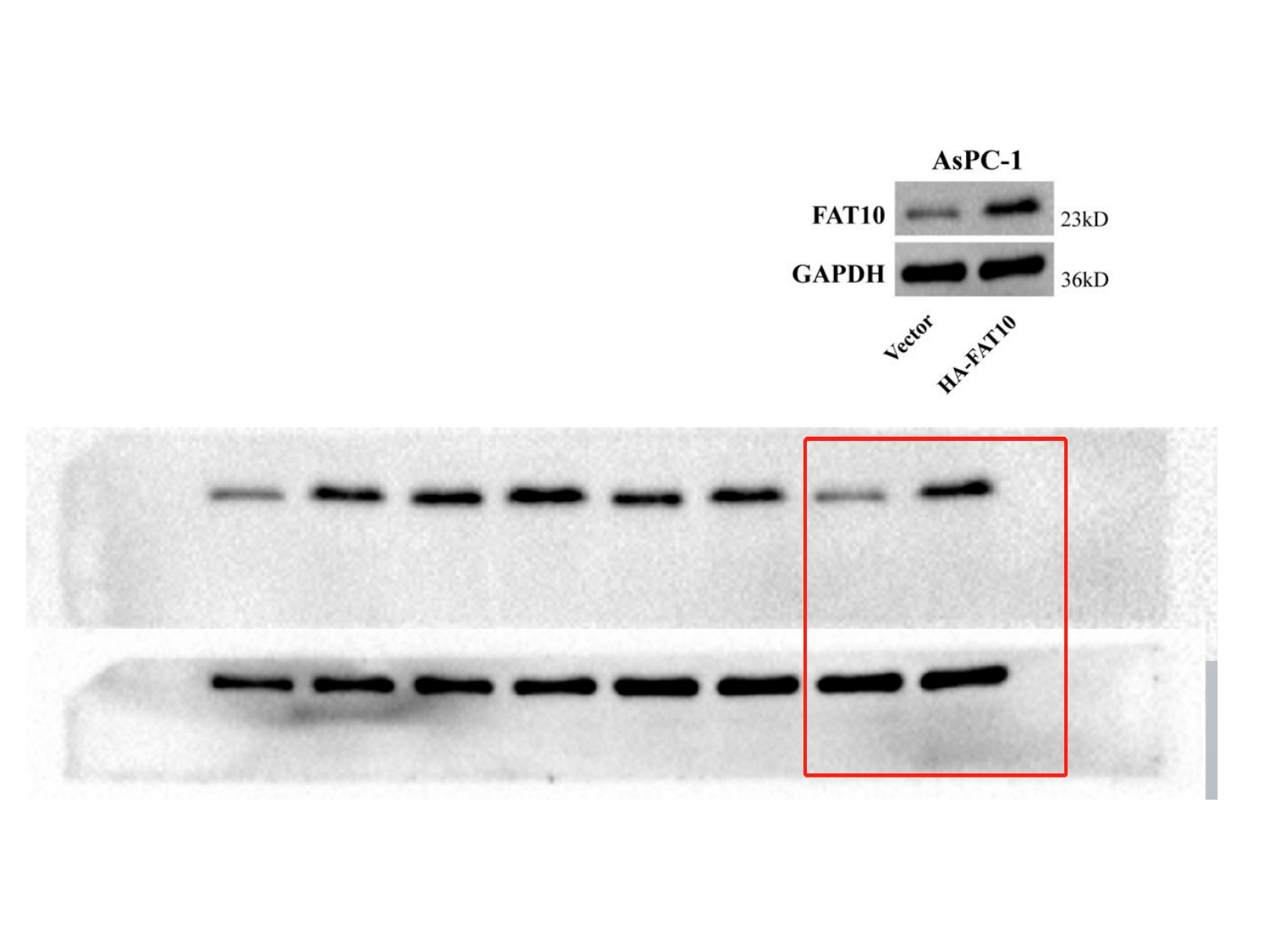

Supplement: Supplementary file 9 — Original Data File [file 41419_2022_4960_MOESM9_ESM.ppt]
